# Supplementary material for: Gene profiles of peripheral white blood cells as potential predictors of pregnancy in embryo-recipient heifers
Source: PLoS One. 2025 Sep 15;20(9):e0330701. doi: 10.1371/journal.pone.0330701 (PMC12435678; doi:10.1371/journal.pone.0330701)
Supplement: Attachment — Submitted filename: PONE-D-25-21659_reviewer.pdf [file pone.0330701.s014.pdf]

PLOS ONE

Gene profiles of peripheral white blood cells as potential predictors of pregnancy in embryo-recipient heifers

--Manuscript Draft--

**Manuscript Number:**	PONE-D-25-21659
**Article Type:**	Research Article
**Full Title:**	Gene profiles of peripheral white blood cells as potential predictors of pregnancy in embryo-recipient heifers
**Short Title:**	Blood cell gene expression as potential predictors of pregnancy success in cattle
**Corresponding Author:**	Mariam Raliou, Ph.D<br>INRAE: Institut National de Recherche pour l'Agriculture l'Alimentation et l'Environnement<br>Jouy-enJosas, Ile de France FRANCE
**Keywords:**	Embryo transfer, heifer, estrous cycle, peripheral white blood cells, PAXgene Blood RNA tubes, biomarkers, pregnancy, microarray, transcriptomic, inflammation, immunity
**Abstract:**	The bovine endometrium undergoes cellular, molecular, and functional changes to support embryo survival and development to term. These changes involve a finely coordinated series of events at both local and systemic levels. We postulated that circulating white blood cells (WBCs) may provide valuable biomarkers to predict pregnancy success in heifers when embryo transfer is used sampled from heifers during a preconceptional cycle and a conception cycle before embryo transfer. WBCs were isolated using PAXgene Blood RNA tubes collected from heifers on Days 7 and 14 of a preconception estrous cycle (PCD7 and PCD14) and, after a rest cycle, on Day 7 of the subsequent estrous cycle (1ETD7) just before embryo transfer. Circulating progesterone and oestradiol-17b were assayed and pregnancy was confirmed by either the delivery of a healthy calf or uterine flushing and conceptus collection on Day 18 post-estrous. Using a bovine oligo-array representing 19,479 unique transcript, comparison of transcriptomes between heifers classified as non-pregnant or pregnant revealed 1,240, 896 and 1,023 differentially expressed genes (DEG) at PCD7, PCD14 and 1ETD7 respectively. Our bioinformatics analyses revealed that pregnancy failure after embryo transfer was associated with upstream regulators, biological functions, canonical pathways and gene networks related to inflammation, immunity, apoptosis and cell death regulation, cell proliferation, membrane compounds, lipid metabolism, oxygen transport and ions transport. The heifers classified as non-pregnant showed significant increased transcripts levels of PTGR1 at the three time points (PCD7, PCD14 and 1ETD7), AIF1 at PCD14, FNDC3B, IL15 and SERPINE1 at 1ETD7. Our findings highlight the potential of peripheral WBCs as a non-invasive source of biomarkers for predicting pregnancy outcomes, offering promising insights for improving pregnancy success when reproductive biotechnologies are used in mammalian females.
**Order of Authors:**	Mariam Raliou, Ph.D
	Marie Margarete Meyerholz
	Doulaye Dembélé
	Kirsten Mense
	Christophe Richard
	Pascale Chavatte-Palmer
	Isabelle Dieuzy-Labaye
	Hans-Joachim Schuberth
	Iain Martin Sheldon, Professor Emeritus
	Olivier Sandra
	Peter Zieger

Powered by Editorial Manager® and ProduXion Manager® from Aries Systems Corporation

	Maike Heppelmann
	Marion Schmicke
**Additional Information:**	
**Question**	**Response**
**Financial Disclosure**<br><br>Enter a financial disclosure statement that describes the sources of funding for the work included in this submission. Review the submission guidelines for detailed requirements. View published research articles from *PLOS ONE* for specific examples.<br><br>This statement is required for submission and **will appear in the published article** if the submission is accepted. Please make sure it is accurate.<br><br>**Funded studies**<br>Enter a statement with the following details:<br>• Initials of the authors who received each award<br>• Grant numbers awarded to each author<br>• The full name of each funder<br>• URL of each funder website<br>• Did the sponsors or funders play any role in the study design, data collection and analysis, decision to publish, or preparation of the manuscript?<br><br>Did you receive funding for this work?	Yes
Please add funding details.<br>as follow-up to "**Financial Disclosure**<br><br>Enter a financial disclosure statement that describes the sources of funding for the work included in this submission. Review the submission guidelines for detailed requirements. View published research articles from *PLOS ONE* for specific examples.<br><br>This statement is required for submission and **will appear in the published article** if the submission is accepted. Please make	The study was funded by the ERA-NET EMIDA (Coordination of European Research on Emerging and Major Infectious Diseases of Livestock), 2010. IMS was supported by the UK Biotechnology and Biological Sciences Research Council Grant BB/1017240/1.

sure it is accurate.<br><br>**Funded studies**<br>Enter a statement with the following details:<br>• Initials of the authors who received each award<br>• Grant numbers awarded to each author<br>• The full name of each funder<br>• URL of each funder website<br>• Did the sponsors or funders play any role in the study design, data collection and analysis, decision to publish, or preparation of the manuscript?<br><br>Did you receive funding for this work?"	
Please select the country of your main research funder (please select carefully as in some cases this is used in fee calculation).<br>as follow-up to "**Financial Disclosure**<br><br>Enter a financial disclosure statement that describes the sources of funding for the work included in this submission. Review the submission guidelines for detailed requirements. View published research articles from *PLOS ONE* for specific examples.<br><br>This statement is required for submission and **will appear in the published article** if the submission is accepted. Please make sure it is accurate.<br><br>**Funded studies**<br>Enter a statement with the following details:<br>• Initials of the authors who received each award<br>• Grant numbers awarded to each author<br>• The full name of each funder<br>• URL of each funder website<br>• Did the sponsors or funders play any role in the study design, data collection and analysis, decision to publish, or preparation of the manuscript?<br><br>Did you receive funding for this work?"	FRANCE - FR

**Competing Interests**<br><br>Use the instructions below to enter a competing interest statement for this submission. On behalf of all authors, disclose any competing interests that could be perceived to bias this work—acknowledging all financial support and any other relevant financial or non-financial competing interests.<br><br>This statement is required for submission and **will appear in the published article** if the submission is accepted. Please make sure it is accurate and that any funding sources listed in your Funding Information later in the submission form are also declared in your Financial Disclosure statement.<br><br>View published research articles from *PLOS ONE* for specific examples.<br><br>**NO authors have competing interests**<br><br>Enter: *The authors have declared that no competing interests exist.*<br><br>**Authors with competing interests**<br><br>Enter competing interest details beginning with this statement:<br><br>*I have read the journal's policy and the authors of this manuscript have the following competing interests: [insert competing interests here]*<br><br>* typeset	The authors have declared that no competing interests exist.
**Ethics Statement**<br><br>Enter an ethics statement for this submission. This statement is required if the study involved:<br><br>• Human participants<br>• Human specimens or tissue<br>• Vertebrate animals or cephalopods	The study was conducted at the clinic for Cattle, University of Veterinary Medicine, Foundation, Hanover with the authorization of the German legislation on animal welfare (Lower Saxony Federal State Office for Consumer Protection and Food Safety, AZ 33.14-42502-04-12/0744). All procedures involving animals were carried out in accordance with German legislation on animal welfare (“Tierschutzgesetz”, https://www.gesetze-im- internet.de/tierschg/BJNR012770972.html).

Powered by Editorial Manager® and ProduXion Manager® from Aries Systems Corporation

• Vertebrate embryos or tissues<br>• Field research<br><br>Write "N/A" if the submission does not require an ethics statement.<br><br>General guidance is provided below. Consult the submission guidelines for detailed instructions. **Make sure that all information entered here is included in the Methods section of the manuscript.**	

Powered by Editorial Manager® and ProduXion Manager® from Aries Systems Corporation

**Format for specific study types**<br><br>**Human Subject Research (involving human participants and/or tissue)**<br>• Give the name of the institutional review board or ethics committee that approved the study<br>• Include the approval number and/or a statement indicating approval of this research<br>• Indicate the form of consent obtained (written/oral) or the reason that consent was not obtained (e.g. the data were analyzed anonymously)<br><br>**Animal Research (involving vertebrate animals, embryos or tissues)**<br>• Provide the name of the Institutional Animal Care and Use Committee (IACUC) or other relevant ethics board that reviewed the study protocol, and indicate whether they approved this research or granted a formal waiver of ethical approval<br>• Include an approval number if one was obtained<br>• If the study involved *non-human primates*, add *additional details* about animal welfare and steps taken to ameliorate suffering<br>• If anesthesia, euthanasia, or any kind of animal sacrifice is part of the study, include briefly which substances and/or methods were applied<br><br>**Field Research**<br><br>Include the following details if this study involves the collection of plant, animal, or other materials from a natural setting:<br>• Field permit number<br>• Name of the institution or relevant body that granted permission	
**Data Availability**<br><br>Authors are required to make all data underlying the findings described fully available, without restriction, and from the time of publication. PLOS allows rare exceptions to address legal and ethical concerns. See the PLOS Data Policy and FAQ for detailed information.	Yes - all data are fully available without restriction

A Data Availability Statement describing where the data can be found is required at submission. Your answers to this question constitute the Data Availability Statement and **will be published in the article**, if accepted.<br><br>**Important:** Stating 'data available on request from the author' is not sufficient. If your data are only available upon request, select 'No' for the first question and explain your exceptional situation in the text box.<br><br>Do the authors confirm that all data underlying the findings described in their manuscript are fully available without restriction?	
**Describe where the data may be found in full sentences. If you are copying our sample text, replace any instances of XXX with the appropriate details.**<br><br>• If the data are **held or will be held in a public repository**, include URLs, accession numbers or DOIs. If this information will only be available after acceptance, indicate this by ticking the box below. For example: *All XXX files are available from the XXX database (accession number(s) XXX, XXX.).*<br>• If the data are all contained **within the manuscript and/or Supporting Information files**, enter the following: *All relevant data are within the manuscript and its Supporting Information files.*<br>• If neither of these applies but you are able to provide **details of access elsewhere**, with or without limitations, please do so. For example:<br><br>*Data cannot be shared publicly because of [XXX]. Data are available from the XXX Institutional Data Access / Ethics Committee (contact via XXX) for researchers who meet the criteria for access to confidential data.*<br><br>*The data underlying the results presented in the study are available from (include the name of the third party*	All transcriptomic profile data files are available in the GEO database (accession number(s): GSE185325) and will be accessible after pubication of the article.

and contact information or URL).<br>• This text is appropriate if the data are owned by a third party and authors do not have permission to share the data.<br><br>* typeset	
Additional data availability information:	Tick here if the URLs/accession numbers/DOIs will be available only after acceptance of the manuscript for publication so that we can ensure their inclusion before publication.

Powered by Editorial Manager® and ProduXion Manager® from Aries Systems Corporation

Manuscript

Click here to access/download;Manuscript;20250421_Manuscript_Raliou_M

Gene profiles of peripheral white blood cells as potential predictors of pregnancy in embryo-recipient heifers

Mariam Raliou[1,2*], Marie Margarete Meyerholz[3,4,] Doulaye Dembélé[5], Kirsten Mense[3,6], Maike Heppelmann[3], Christophe Richard[1,2], Pascale Chavatte-Palmer[1,2], Isabelle Dieuzy-Labaye[7#], , Peter Zieger[8], Hans-Joachim Schuberth[9], Marion Schmicke[3,], Iain Martin Sheldon[10] & Olivier Sandra[1,2*]

[1]Université Paris-Saclay, UVSQ, INRAE, BREED, Jouy-en-Josas, France

[2]Ecole Nationale Vétérinaire d'Alfort, BREED, Maisons-Alfort, France

[3]University of Veterinary Medicine Hannover, Foundation, Clinic for Cattle, Endocrinology Laboratory, Bischofsholer Damm 15, Hannover, Germany;

[4]Clinic for Ruminants with Ambulatory and Herd Health Services, Centre for Clinical Veterinary Medicine, Ludwig-Maximilians-University Munich, Sonnenstrasse 16, Oberschleißheim, Germany

[5]Institut de Génétique et de Biologie Moléculaire et Cellulaire, CNRS UMR 7104 - Inserm U 964 - Université de Strasbourg, 1 rue Laurent Fries, Illkirch, France ;

[6]Masterrind GmbH, Osterkrug 20, Verden, Germany

[7]Zoetis, Mercuriusstraat 20, Zaventem, Belgium;

[8]Förster-Technik GmbH, Gerwigstrasse 25, Engen, Germany;

[9]Institute of Immunology, University of Veterinary Medicine, Foundation, Hannover, Buenteweg 2, Hannover, Germany;

[10]Swansea University Medical School, Swansea University, Singleton Park, Swansea, UK

***: Current address** « OIE Organisation Mondiale de la Santé Animale, 12, rue de Prony 75017 Paris, France) »*

* Corresponding authors

E-mail: mariam.raliou@inrae.fr (MR); ORCID ID: 0000-0002-3828-1421

E-mail: olivier.sandra@inrae.fr (OS); ORCID ID: 0000-0001-6957-2670

Financial Disclosure: The study was funded by the ERA-NET EMIDA (Coordination of European Research on Emerging and Major Infectious Diseases of Livestock), 2010. IMS was supported by the UK Biotechnology and Biological Sciences Research Council Grant BB/1017240/1.

Running title

Blood cell gene expression as potential predictors of pregnancy success in cattle

Abstract

The bovine endometrium undergoes cellular, molecular, and functional changes to support embryo survival and development to term. These changes involve a finely coordinated series of events at both local and systemic levels. We postulated that circulating white blood cells (WBCs) may provide valuable biomarkers to predict pregnancy success in heifers when embryo transfer is used sampled from heifers during a preconception cycle and a conception cycle before embryo transfer. WBCs were isolated using PAXgene Blood RNA tubes collected from heifers on Days 7 and 14 of a preconception estrous cycle (PCD7 and PCD14) and, after a rest cycle, on Day 7 of the subsequent estrous cycle (1ETD7) just before embryo transfer. Circulating progesterone and oestradiol-17b were assayed and pregnancy was confirmed by either the delivery of a healthy calf or uterine flushing and conceptus collection on Day 18 post-estrus. Using a custom bovine gene expression microarray representing 19,479 unique transcript, comparison of transcriptomes between heifers classified as non-pregnant or pregnant revealed 1,240, 896 and 1,023 differentially expressed genes (DEGs) at PCD7, PCD14 and 1ETD7 respectively. Our bioinformatics analyses revealed that pregnancy failure after embryo transfer was associated with upstream regulators, biological functions, canonical pathways and gene networks related to inflammation, immunity, apoptosis and cell death regulation, cell proliferation, membrane compounds, lipid metabolism, oxygen transport and ions transport. The heifers classified as non-pregnant showed significant increased transcripts levels of *PTGR1*

at the three time points (PCD7, PCD14 and 1ETD7), *AIF1* at PCD14, *FNDC3B*, *IL15* and *SERPINE1* at 1ETD7. Our findings highlight the potential of peripheral WBCs as a non-invasive source of biomarkers for predicting pregnancy outcomes, offering promising insights for improving pregnancy success when reproductive biotechnologies are used in mammalian females.

Summary Sentence

This study indicates that transcriptomic changes in WBCs during the estrous cycle may serve as predictive biomarkers for embryo transfer success in recipient females.

Keywords: embryo transfer, heifer, estrous cycle, peripheral white blood cells, PAXgene Blood RNA tubes, biomarkers, pregnancy, microarray, transcriptomic, inflammation, immunity

INTRODUCTION

In mammals, successful implantation requires finely coordinated paracrine and endocrine signaling between the embryo and the receptive uterus, leading to significant physiological and molecular changes in the maternal endometrium, including immune system modulation (1-3). Circulating immune cells are recruited to the uterus, where they facilitate interactions between the maternal environment and the early conceptus by preventing infections, eliminating apoptotic cells, promoting immunotolerance, and supporting conceptus development (4, 5). In response to the presence of the embryo, the regulation of immune cells such as monocytes, macrophages, and dendritic cells triggers the upregulation of cytokines like IL-12B and IL-15, and a downregulation of IL-18 in the bovine endometrium (6). Trophectoderm cells of the conceptus secrete interferon-tau (IFNT), which is the key signal for pregnancy recognition in

ruminants. IFNT maintains progesterone production by inhibiting luteolysis and modulates the maternal immune response (7, 8).

During the peri-implantation period, IFNT-stimulated genes are significantly elevated in the plasma of pregnant cows, while pro-inflammatory cytokines (such as IL-8 and TNF-α) and adhesion molecules (like CD62L and CD11b) show higher expression in non-pregnant cows (2). In women, studies suggest that immune cells, in concert with the endocrine system, facilitate early embryo-maternal interactions (9). Interestingly, lymphocyte subpopulations such as CD56+ and CD16+CD56+ cells are more abundant in the peripheral blood of women who experience implantation failure compared to those with successful implantation (10). These findings underscore the critical role of circulating immune cells in preparing the maternal environment for embryo survival and pregnancy maintenance.

The endometrium acts as a biological sensor and regulator of pregnancy success, influencing embryo development and adapting to long-term changes (11, 12). Considering the close interactions between the endometrium and circulating immune cells (13), systemic phenotyping of immune cell profiles could help predict a female's ability to become and remain pregnant (14). For example, transcriptome profiles of peripheral blood mononuclear cells (PBMCs) collected before artificial insemination correlate with fertility in beef heifers (3). Intrauterine administration of autologous PBMCs during the estrous cycle has been shown to improve pregnancy rates following bovine embryo transfer (15). Similarly, the intrauterine administration of PBMCs from non-pregnant mice before embryo implantation enhanced endometrial receptivity and implantation in mice with implantation dysfunction (16). Furthermore, PBMC administration in women with repeated in vitro fertilization (IVF) failure has been reported to improve embryo implantation (17) and in women with repeated implantation failure (RIF), PBMCs decrease the expression of estrogen receptor α (ERα) and

progesterone receptor (PR), enhancing endometrial receptivity during the implantation window (18).

During gestation, the endometrium undergoes changes in immune cell populations, with increases in CD14(+) and CD172a-CD11c(+) cells and elevated expression of cytokine genes such as IL-12B and IL-15, while IL-8 transcript levels decrease (6). Increased levels of inflammatory cytokines such as TNF-α and IFN-γ in PBMCs are associated with infertility (19). Among the cytokines and immune cell-attracting chemokines, IL-15 plays a key role in recruiting natural killer cells to the endometrium, where they differentiate into uterine natural killer cells, aiding implantation in both mice and humans (20). These findings demonstrate the importance of cytokines and immune cells in mediating communication between the uterus and the peripheral immune system.

The corpus luteum plays a crucial role in regulating the immune system to create a favorable maternal environment for pregnancy (21). Steroid hormones regulate the production of growth factors, cytokines, lipid mediators, and transcription factors in the endometrium (22). In the bovine endometrium, progesterone and estrogen are key regulators of gene expression related to immunity, angiogenesis, tissue homeostasis, metabolism, and transport processes (23, 24). A recent study found that variations in the expression of bovine endometrial genes related to extracellular matrix interactions, histotroph composition, prostaglandin synthesis, TGF signaling, inflammation, and leukocyte activation on Day 7 of the estrous cycle are crucial for conception (25). Similarly, changes in immune cells and cytokine gene expression in the human endometrium are essential for successful conception (26). These findings collectively highlight the vital role of immune cells in preparing the uterus for pregnancy.

Circulating immune cells are influenced by the physiological state of the uterus and may serve as indicators of a female's ability to support pregnancy to term. In cattle, early embryonic mortality, often occurring between 8 and 16 days post-insemination, reduces production

efficiency (27). Early embryonic loss during the pre-implantation period is often linked to a compromised endometrial environment or embryonic incompetence (28). In the present study, we hypothesize that gene expression in peripheral white blood cells (WBC) during the estrous cycle may predict a female's capacity to initiate and sustain pregnancy after embryo transfer. The mRNA profiles were analyzed from WBCs of heifers that did not achieve pregnancy after two successive embryo transfers (non-pregnant heifers) and those that became pregnant after the first transfer (pregnant heifers). Samples were collected on Days 7 and 14 post-estrus during the preconception cycle, as well as on Day 7 post-estrus during the conception cycle.

Materials and Methods

Ethics Statement

The study was conducted at the clinic for Cattle, University of Veterinary Medicine, Foundation, Hanover with the authorization of the German legislation on animal welfare (Lower Saxony Federal State Office for Consumer Protection and Food Safety, AZ 33.14-42502-04-12/0744). All procedures involving animals were carried out in accordance with German legislation on animal welfare (“Tierschutzgesetz”, https://www.gesetze-im-internet.de/tierschg/BJNR012770972.html).

Animal experiments

Thirty Holstein Friesian heifers (Masterrind, Germany) with a mean age of 14.1 ± 1.6 months and a mean body weight of 337.8 ± 23.7 kg at the start of the experiment were purchased from a single farm in Denmark. Prior to the initiation of the experiment, the animals underwent a two-week adaptation period. The conditions for the maintenance and monitoring of the animals were previously described by Meyerholz et al. (29).

The experimental protocol is summarized in Fig 1. Animals underwent serial estrus synchronization and two embryo transfers (ET). Synchronization involved an intramuscular injection of 2 ml prostaglandin F2α (Estrumate; Intervet, Germany), followed by 2 ml gonadotropin-releasing hormone (Receptal; Intervet, Germany) 48 hours later to induce ovulation. Ovulation was monitored twice daily by transrectal ultrasound (LOGIQ Book XP, linear transducer probe 10 MHz; General Electric Medical Systems, China). If ovulation was not detected, the synchronization protocol was repeated.

After the first preconception synchronization cycle, animals underwent two consecutive synchronization cycles with embryo transfer (ET). On Day 7 post-estrus, ET was performed in suitable recipients with a corpus luteum > 1.5 cm and serum progesterone > 1.0 ng/ml (Immulite System, Siemens Healthcare Diagnostics, USA), as previously described (30). All transferred embryos were male, produced, graded 2+ or 2-, and sexed by Masterrind GmbH (Verden, Germany). Embryos were frozen in glycerol and stored in liquid nitrogen, then thawed under sterile conditions using the Emcare Embryo Thawing System (ICPbio Reproduction, USA). One embryo was transferred trans-cervically using an embryo transfer instrument (Transfer Stylet 19240/1000, Transfit Lateral 19240/1005, Sanitary Sheaths 19271/0080; Minitüb, Germany).

Fig 1. Overview of the experimental procedures and heifer phenotypes

(**Upper panel**): Estrus synchronization without embryo transfer defined the preconception cycle (PC). After completing the PC, females were left undisturbed for one month before undergoing a second estrus synchronization, this time combined with the transfer of a single male embryo on Day 7 post-estrus. Heifers in this conception cycle (1ET) were either carried to term pregnancy or subjected to uterine flushing on Day 18 post-estrus to recover the conceptus. Following uterine flushing, the females were again left for one month before entering a third estrous synchronization, with the transfer of another embryo on Day 7 post-estrus. At Day 18 of this second conception cycle, pregnancy phenotype was

assessed via uterine flushing. For each cycle, blood samples were collected on Days 7 (D7), 14 (D14), and 18 (D18) to measure circulating progesterone (P4) levels. For microarray analyses, peripheral blood was collected using PAXgene blood RNA tubes on D7 and D14 of the PC, as well as on D7 of the 1ET cycle (1ETD7). (**Lower panel**): For each cycle, numbers of heifers and their phenotype is indicated.

Pregnancy Detection

Pregnancy success was determined on Day 18 post-estrus through circulating progesterone measurement, uterine flushing, and conceptus collection, as described previously (31). Animals were considered pregnant if circulating progesterone (P4) ≥ 1 ng/ml and an elongated blastocyst with an embryonic disc was present. Non-pregnant females had P4 < 1 ng/ml and no conceptus flushed. Following the flushing, non-pregnant animals received a 2 ml injection of prostaglandin F2α to induce a physiological estrous cycle and were rested for 1 month before a second synchronization and embryo transfer. Four pregnant females from the first ET were allowed to carry to term and were classified as pregnant (n = 4). Heifers failing to conceive after two consecutive embryo transfers were classified as non-pregnant (n = 9). Animals with discordant results (P4 ≥ 1 ng/ml but no conceptus collected; n = 1 in the first estrous cycle; n = 3 in the second) and with embryonic mortality (n = 2) were excluded from further analysis.

Progesterone and estradiol-17 beta assays

Serum progesterone and estradiol-17 beta concentration were determined in heifers categorized as non-pregnant (n = 9) and pregnant (n = 4), as previously described (30, 32) and as shown in Table 1. The serum levels of progesterone were measured with a solid-phase radioimmunoassay (TKPG1, Coat-a-count Progesterone; Siemens Diagnostics, USA) and the concentrations of serum estradiol-17 beta were determined using a direct enzyme-immunoassay (EIA).

Table 1: **Details of Holstein Friesian heifers used for serum progesterone and Estradiol-17 beta assays, microarray hybridization and RT-qPCR validation**.

Animal ID (Heifer: H)	Physiological status of animals after two embryo transfert	**plasma progesterone and estradiol-17 beta assay**			**Microarray hybridation/analyses**			**RT-PCR validation**		
		Preconception cycle /days post-estrus (PCD)		First embryo transfer/days post-estrus (1ETD)	Preconception cycle /days post-estrus (PCD)		First embryo transfer/days post-estrus (1ETD)	Preconception cycle /days post-estrus (PCD)		First embryo transfer/days post-estrus
		PCD7	PCD14	1ETD7	PCD7	PCD14	1ETD7	PCD7	PCD14	1ETD7
H2	**Non-pregnant (n = 9)**	X	X	X			X	X	X	X
H12		X	X	X			X	X	X	X
H13		X	X	X	X	X	X	X	X	X
H14		X	X	X	X	X	X	X	X	X
H15		X	X	X	X	X	X	X	X	X
H18		X	X	X	X	X		X	X	
H21		X	X	X	X	X		X	X	
H23		X	X	X	X	X		X	X	
H32		X	X	X	X	X		X	X	
H1	**Pregnant (n = 4)**	X	X	X	X	X	X	X	X	X
H11		X	X	X	X	X	X	X	X	X
H19		X	X	X	X	X	X	X	X	X
H22		X	X	X	X	X		X	X	X

Holstein Friesian heifers underwent 3 cycles of estrus synchronization. The first cycle was devoid of embryo transfer (preconception cycle: PC). The second and third cycles were associated with embryo transfer on Day 7 (1EDT) post-estrus (conception cycles 1 and 2). Females were classified as non-pregnant if pregnancy was undetectable on Day 18 post-estrus at conception cycles 1 and 2. Females were classified as pregnant when the term of pregnancy was reached at conception cycle 1 or when embryo was recovered on Day 18 post-estrus at conception cycles 1 and 2. X: biochemical and/or molecular analyses run with blood sampling.

PAXgene blood sample collection and processing

Two PAXgene Blood RNA Tubes (BD, Germany; 2.5 ml/tube) were collected per female. For each tube, 2.5 ml of whole blood was drawn from the jugular vein on Day 7 (PCD7) and Day 14 (PCD14) of the preconception cycle, as well as on Day 7 just before the first embryo transfer (1ETD7).

Total RNA extraction and purification

Total RNA was isolated using the PAXgene Blood RNA system (PreAnalytiX, Qiagen/BD Company, France) according to the manufacturer's instructions, modified as described (33). The quality and integrity of the isolated total RNA were assessed using the Agilent 2100 Bioanalyzer (@bridge ICE -Iso Cell Express-, INRA, France: https://www6.jouy.inra.fr/ice). The RNA integrity number (RIN) for each sample ranged from 7 to 9.5.

Microarray hybridization

Transcriptional profiling was conducted using a custom 8x60K bovine gene expression microarray, designed based on annotated bovine Ensembl transcripts (http://www.ensembl.org/index.html, genome assembly UMD3.1) represented 19,479 transcripts, previously as described (33). RNA labeling was done with total RNA isolated from blood collected from 7 females classified as non-pregnant and 4 pregnant at PCD7 and PCD14. Due to blood sampling issues, at 1ETD7, microarray hybridization was carried out on total RNA from WBCs collected from 5 non-pregnant and 3 pregnant animals (Table 1). RNA labeling followed the Agilent "One-Color Microarray-Based Gene Expression Analysis" protocol (33). The microarray data were submitted to the GEO database (accession number GSE185325). Differentially expressed genes (DEGs) were identified using the fold change rank ordering (FCROS) method (34) and t-test with the TREAT option of the LIMMA method (35), comparing non-pregnant with pregnant females. p-values were adjusted using the Benjamini & Hochberg method (36), and a threshold was applied to select significant DEGs.

Principal Component Analysis

Principal component analysis (PCA) was performed using the FactoMineR package (37) to assess sample dispersion, first using log2-transformed counts of all genes, then only the DEGs.

Confidence ellipses around categories represent the means with a 0.95 confidence level, based on empirical variance divided by the number of observations.

Ingenuity pathway and interaction network analysis

Ingenuity pathway analysis was performed to identify associations between the differentially expressed genes (DEGs) from peripheral white blood cells (WBCs) and canonical pathways, upstream regulators as well as and gene networks (IPA; Ingenuity® Systems, www.ingenuity.com). The DEGs from PCD7, PDC14, and 1ETD7 were analyzed using a right-tailed Fisher exact test. The analysis included overrepresented canonical pathways, integration of these pathways into biological networks, and predicted of upstream regulators. An interaction network was generated for each DEG list. Additionally canonical pathways and functions were analyzed using the DAVID Bioinformatics Resources interface. Functions and pathways with p-values < 0.05 were considered significant.

Real-time quantitative PCR (qPCR) analysis

For RT-qPCR (Reverse Transcription quantitative Polymerase Chain Reaction) quantification, the following number of heifers was used: nine females classified as non-pregnant at PCD7 and PCD14, five non-pregnant at 1ETD7; five pregnant females at PCD7 and PCD14, and three pregnant females at 1ETD7. RT-qPCR was performed on 12 differentially expressed genes identified in WBCs from both non-pregnant and pregnant females as described (33). RNA expression levels were normalized using three stable reference genes (*ACTB, GAPDH* and *RLP19*), whose stability was evaluated using the geNorm module of Qbase software (Biogazelle) (38). Gene expression data are presented as mean calibrated normalized relative quantity (CNRQ) values in arbitrary units.

Statistical analysis

Statistical analyses of RT-qPCR data were performed using Prism 9.0.0 (GraphPad, USA). Data normality was tested with D'Agostino & Pearson and Shapiro-Wilk tests. Gene expression differences between non-pregnant and pregnant animals at PCD7, PCD14, and 1ETD7 were analyzed using repeated measures two-way ANOVA or mixed-effects model with Geisser-Greenhouse correction, Sidak's multiple comparisons test ($p \leq 0.05$).

RESULTS

Pregnancy rates after embryo transfers

All embryos used in this study are from different sires. Upon first embryo transfer (1ET), diagnosis of pregnancy was based on circulating progesterone concentration (P4 > 1ng/ml or P4 < 1 ng/ml) measured at day 18 post-estrus (Fig 1). Out of 30 Holstein Friesian heifers, 10 were diagnosed pregnant. Four of them were left for completing pregnancy to term and uterine flushing was used for the six remaining heifers, leading to the recovery of an elongated conceptus with an embryonic disc. Among the 26 heifers, including 6 initially classified as pregnant and 20 as non-pregnant, 10 conceived after the second embryo transfer, including one that became pregnant following two consecutive transfers. Conversely, 16 heifers remained non-pregnant, with 9 failing to establish pregnancy after both embryo transfers. Accordingly, pregnancy rates (Fig 1) was 33.3% for the first embryo transfer and 36.0% for the second embryo transfer. During the experimental protocol, no significant difference in weight gain was observed for any of the 30 females (data not shown). Analyses were performed on heifers that were allowed to carry pregnancies to term, including one heifer diagnosed as pregnant twice at Day 18 post-embryo transfer, and on heifers that remained non-pregnant after two consecutive

rounds of embryo transfer. These groups were subsequently referred to as "pregnant" and "non-pregnant" females in the following analyses (S1 Table).

Changes in serum steroid hormones

On Days 7 and 14 post-estrus of the preconception cycle (PCD7, PCD14), circulating progesterone concentrations (P4) were significantly higher in non-pregnant heifers (n =7) than in the pregnant 4 heifers (Fig 2A). Upon embryo transfer, P4 concentrations were significantly higher in pregnant heifers than non-pregnant animals at Day 18 (Fig 2C). Estradiol-17 beta concentrations did not differ significantly between the groups (Fig 2B and 2D).

Fig 2. Progesterone and estradiol-17 beta concentrations (ng/ml) on days 7, 14 and 18 post-estrus in Holstein Friesian heifers. Blood was collected from Holstein Friesian heifers, which were subsequently classified as pregnant (n = 4) or non-pregnant (n = 9) based on pregnancy diagnosis, determined by circulating P4 levels and embryo recovery on Day 18 post-embryo transfer. Females were sampled on Days 7, 14 and 18 post-estrus during the preconception cycle (A, B) and the first conception cycle (C, D).

Transcriptomic profiling and analysis

Using a custom bovine gene expression array containing 19,479 unique transcripts- element bovine oligonucleotide array (33), gene expression profile were determined using peripheral white blood cells (WBCs) collected at Day 7 post-estrus (PCD7) and Day 14 post-estrus (PCD14) of the preconception cycle as well as Day 7 of the cycle associated with embryo transfer (1ETD7). Based on RNA samples, transcriptomes were analyzed in 7 heifers classified as non-pregnant and 4 heifers classified as pregnant at PCD7 and PCD14 as well as 5 out of 7 heifers of the non-pregnant group and 3 out of the 4 females of the pregnant group at 1ETD7.

The fourth heifer, diagnosed as pregnant after the first embryo transfer and allowed to carry the pregnancy to term, was excluded from the analysis due to blood collection occurring after the embryo transfer. Therefore, the heifer that became pregnant following two successive embryo transfers was included in the pregnant group (S1 Table). Volcano plots that combined fold change (FC) and probabilities (f-value ≤ 0.015 for low-expressed probes and f-value ≥ 0.985 for over-expressed probes) from the fold change rank ordering statistics (FCROS) method (34) were used to visualize DEGs distribution between females of the non-pregnant group and of the pregnant group at Days PCD7, PCD14 and 1ETD7 (Fig 3A–C). The low-expressed and over-expressed probes were indicated in green and red color respectively. Using TREAT-option of the LIMMA statistical method (t-test relative to a threshold, p-value < 0.05 and FDR $\geq$ 10 %; FC $\geq$ 1.2 or FC $\leq$ 0.80), we identified 1240 DEGs, 896 DEGs and 1023 DEGs between the females classified as non-pregnant and those classified pregnant group at PCD7, PDC14 and 1ETD7 respectively.

At PCD7, expression levels of 550 DEGs and 690 DEGs were higher and lower respectively in WBCs from females classified as non-pregnant group when compared with females of the pregnant group (S2 Table). At PCD14, expression levels of 417 DEGs and 479 DEGs were higher and lower respectively in the WBCs from females of the non-pregnant group when compared with females of the pregnant group (S3 Table). At 1ETD7, expression levels of 532 DEG and 491 DEGs in WBCs were higher and lower respectively in the non-pregnant vs. pregnant females comparison (S4 Table). Based on the DEGs, principal component analyses showed a separation between the WBCs transcriptomic profiles of the non-pregnant and pregnant animals at PCD7, PCD14 or 1ETD7 (Fig 3D–F).

Fig 3. Volcano plots and Principal component analysis (PCA) showing the association between gene expression in peripheral white blood cells (WBC) and physiological status of Holstein Friesian heifers.

(A, B, C) Volcano plots of gene expression profiles (all probes) in peripheral white blood cells on Day 7 (PCD7) and 14 (PCD14) of the preconception cycle comparing heifers based on pregnancy outcome at first conception (classified as non-pregnant, n = 7; pregnant, n = 4), as well as on Day 7 of the conception cycle (1ETD7) at the time of embryo transfer (classified as non-pregnant, n = 5; pregnant, n = 3). Each dot represents the mean expression of individual gene obtained from a microarray normalized dataset. The cut-off values (black dotted lines) were established according to the following parameters: |log10(fold change (FC)| ≥ 2. and FDR ≥ 10%. The red dots represent the over-expressed transcripts (f.value ≤ 0.05) and the green dots represent the under-expressed transcripts (f.value ≥ 0.95). Genes above the cut-off lines have been considered as differentially expressed. The y-axis corresponds to the mean expression value of log 10 (p-value), and the x-axis displays the log2 fold change value (females classified as non-pregnant vs. females classified as pregnant at D18 post-estrus following embryo transfer). (**D, E, F**) Principal Component Analysis (PCA) of differentially expressed genes (DEGs) from microarray data of peripheral white blood cells (WBCs) obtained from females classified as non-pregnant (n = 7) and pregnant (n = 4) at PCD7, PCD14, and 1ETD7 (classified as non-pregnant, n = 5; pregnant, n = 3), with a p-value ≤ 0.05

Pregnancy success is associated with distinct gene expression profiles in white blood cells (WBCs)

Venn diagram was used to compare the numbers of genes differentially expressed in WBCs isolated from females classified as non-pregnant and pregnant (Fig 4). Using TREAT, we found that 998 DEGs (FC ≥ 2, n =8), 655 DEGs (FC ≥ 2, n =9) and 847 DEGs (FC ≥ 2, n =15) were specific to PCD7, PCD14, and 1ETD7, respectively. Whereas 132 DEGs were common to PCD7 and PCD14, 77 DEGs were common to PCD7 and 1ETD7and 76 DEGs were common to PCD14 and 1ETD7. A total of 33 DEGs were found to be common between PCD7, PCD14 and 1ETD7 (Fig 4 and S5 Table).

Fig 4. Venn diagrams and Heatmaps of differential gene expression in peripheral white blood cells (WBC) of Holstein Friesian heifers according to the ability to become pregnant. Heatmaps highlighting the Top 100 (50 up-regulated and 50 down-regulated) differentially expressed genes (DEGs) identified in WBCs on Days 7 (PCD7) and 14 (PCD14) of the preconception cycle between heifers according the pregnancy outcome at the first conception cycle (classified as non-pregnant, n =7; pregnant, n = 4) as well as at Day 7 of the conception cycle at the time of embryo transfer (classified as non-pregnant, n =5; pregnant; n = 3). Heatmaps were generated by Cluster 3.2 software and visualized with TheeView software. P-value ≤ 0.05

The heatmaps generated from the Top 100 differentially expressed genes in WBCs from the heifer group that became pregnant and those that were unable to conceive revealed that gene expression patterns were correlated with both the stage of the estrous cycle (PCD7, PCD14, and 1ETD7) and the animals' phenotype (Fig 4). These DEGs are linked to various biological pathways, including immune response and inflammation, cell proliferation and cycle regulation, lipid and phospholipid metabolism, skeletal development and mineralization, as well as development, differentiation, apoptosis, and cell death regulation.

Among the 33 DEGs common to PCD7, PCD14 and 1ETD7, 16 DEGs consistently presented higher expression levels (FC ≥ 1.2 or FC ≤ 0.80, p-value < 0.05) in the WBCs of the heifers classified as non-pregnant, while 10 DEGs consistently exhibited higher expression level in the heifers classified as pregnant. The expression levels of 7 DEGs were variable across PCD7, PCD14 and 1ETD7 (Fig 5 and S5 Table).

Fig 5. Heatmap highlighting 33 common differentially expressed genes (DEGs) at PDC7, PCD14 and 1ETD7. DEGs were identified in peripheral white blood cells (WBCs) from Holstein Friesian heifers classified as non-pregnant (Non-preg.) and compared to those classified as pregnant group (Preg.). Samples were collected on Days 7 and 14 of the preconception cycle and on Day 7 after the first

embryo transfer. A heatmap was generated using Cluster 3.2 software and visualized with TreeView software

Networks associated with differentially expressed genes (DEGs) in WBCs related to pregnancy outcome

The differentially expressed WBC genes between heifers classified as non-pregnant and pregnant were mapped onto a global molecular network using Ingenuity Pathways Analysis (IPA). At PCD7, the IPA network module identified 25 networks, with one of the main networks, “Cell Signaling, Nucleic Acid Metabolism, and Small Molecule Biochemistry” (n = 24 genes, 22 of which are focus genes), linking genes involved in biological functions such as cell signaling, nucleic acid metabolism, and small molecule biochemistry, with a central role played by a G protein-coupled receptor (Fig 6A and S6 Table) in the non-pregnant group. At PCD14, 25 networks were also identified, with the most biologically relevant network related to “Developmental Disorder, Organismal Injury and Abnormalities, Renal Hypoplasia” (n = 28 genes, 23 of which are focus genes, Fig 6B and S7 Table) in the non-pregnant group. This network links genes involved in biological processes that influence organ structure and function. At 1ETD7, among the 25 networks identified, the main network was associated with “Neurological Disease, Cellular Growth and Proliferation, Hematopoiesis” (n = 41 genes, 30 of which are focus genes, Fig 6C and S8 Table) in the non-pregnant group. This network connects genes that regulate nervous system function, orchestrate hormonal and organ responses, and control cell proliferation critical for organ development, preparation, hematopoiesis, and immune function. Ingenuity Pathways Analysis also revealed a central role for fibronectin 1 (FN1) and ubiquitin B (UBB) in organizing this network.

Fig 6. Major networks of transcripts that are associated with the ability of Friesan-Holstein heifers to become pregnant upon embryo transfer. Selected networks (based on) contain up-regulated and down-regulated differentially expressed genes (DEGs) identified in peripheral white blood cells (WBCs) collected from the two groups of Holstein Friesian heifers on Days 7 and 14 of the preconception cycle (PCD7 and PCD14) and at Day 7 of the first cycle with embryo-transfer (1ETD7). Top functions in the selected networks are linked to (A) "Cell Signaling, Nucleic Acid Metabolism, Small Molecule Biochemistry" at PCD7 with (B) "Developmental Disorder, Organismal Injury and Abnormalities, Renal Hypoplasia" at PCD14 and with (C) "Neurological Disease, Cellular Growth and Proliferation, Hematopoiesis" at 1ETD7. Network displays nodes (genes/gene products) and edges (the biological relationship between nodes). Red and green shaded nodes represent up- and down-regulated gene expression, respectively. The color intensity of the nodes indicates the fold change increase (red) or decrease (green) associated with a particular gene. Solid line indicates a direct interaction between nodes (genes/gene products) and a dashed line indicates an indirect relationship between nodes. White symbols indicate neighboring genes that are functionally associated, but not included in the differentially expressed gene list. The shape of the node is indicative of its function (legend available online https://www.qiagenbioinformatics.com/products/ingenuity-pathway-analysis.

Biological functions and canonical pathways linked with differentially expressed genes in WBCs associated with pregnancy outcome

Biological terms and functions associated with differentially expressed WBC genes between females classified as non-pregnant and pregnant were identified using DAVID (Database for Annotation, Visualization and Integrated Discovery). Gene Ontology (GO) and Kyoto Encyclopedia of Genes and Genomes (KEGG) analyses were performed on 1240, 896, and 1023 DEGs identified at PCD7, PCD14 and 1ETD7, respectively. The top five significant terms/functions were presented for each time point in Fig 7. A total of 27, 11, and 28 functional

categories and canonical pathways c ($n \geq 5$ genes by category, p-value ≤ 0.05) were associated with DEGs at PCD7, PCD14 and 1ETD7 respectively (S9 Table).

The major functional category terms and canonical pathways associated with highly expressed genes in heifers classified as non-pregnant were “regulation of growth” and “leukocyte transendothelial migration” at PCD7, “inflammatory response” and “endocytosis and inflammatory response” at PDC14, and “angiogenesis” and “HTLV-I infection” ” at 1ETD7, respectively (Fig 7). In heifers classified as pregnant, DEGs were associated with “microtubule cytoskeleton organization” and Calcium signaling pathway” at PCD7, “nucleic acid binding and positive regulation of angiogenesis” at PCD14, and “GTPase activity and metabolic pathways” at 1ETD7 (Fig 7).

Fig 7. Top five functional categories (GO and KEEG) of genes significantly enriched in peripheral white blood cells collected from Holstein Friesian heifers. Using DAVID tools, analyses were conducted on significantly ($P < 0.05$) up- or down-regulated genes identified between heifers that were classified as non-pregnant ($n = 7$) vs. pregnant ($n = 4$) on Days 7 of the preconception cycle (PCD7, PCD14) as well as on Day 7 of the first conception cycle just before embryo transfer (1ETD7). Bars represent the fold enrichment and number of genes is indicated in brackets. The list of genes under each category is provided in Supplementary Table 9.

Upstream regulators linked with differentially expressed genes in WBCs associated with pregnancy outcome

The upstream analysis module of IPA identified 694, 1,087, and 1,401 upstream regulators (p-value of overlap ≤ 0.05) at PCD7, PCD14, and 1ETD7, respectively (S10-12 Tables), in WBCs collected from heifers classified as non-pregnant compared to those classified as pregnant. Among these, 142 upstream regulators were common across all three-time points (S13 Table),

with 21 showing significant activation or inhibition (Z-score ≥ +2 or ≤ -2, p-value of overlap ≤ 0.05; Table 2). Common upstream regulators, including the growth factor AGT, the transcription factor EGR2, the cytokines IFNG and IL1B, and the chemical compound lipopolysaccharide, which are involved in immune and inflammatory responses, were predicted to be activated. Conversely, the chemical kinase inhibitor PD98059, which plays a role in proliferation, differentiation, and survival; the enzyme VEGF; the chemical compound VEGFA, which is involved in angiogenesis and vascular permeability; and the mature microRNAs miR-155-5p (with the UAAUGCU seed sequence) and miR-16-5p (along with other miRNAs sharing the AGCAGCA seed sequence), which regulate immune responses and inflammation, were predicted to be inhibited (Table 2).

Among the 376 upstream regulators specific to PCD7, the chemical drug (indomethacin), the calcium-binding protein A8 (S100A8) and the transcription regulator (GATA6) were activated, while the mature microRNA (miR-146a-5p (and other miRNAs w/seed GAGAACU)), the chemical - protease inhibitor (N-[N-(3,5-difluorophenacetyl-L-Ala)]-S-phenylglycine t-butyl ester) and the transcription regulator (TAF4) were inhibited. Among the 558 upstream regulators specific to PCD14, the transcription regulators (TP63, POU5F1) and the ligand-dependent nuclear receptor (AR) were activated, while fusion gene/product (ETV6-RUNX1), the enzyme (EGLN) and the ion channel (GRIN3A(were inhibited. Among the 896 upstream regulators specific to 1ETD7, the transcription regulator (STAT4) and the cytokines (PRL, IL1), were activated, while the transcription regulators (CITED2), TSC2 and the phosphatase (PTPRR) were inhibited.

Table 2: Top activated and inhibited upstream regulators in peripheral white blood cells (WBCs) common to the preconception cycle (Days 7 and 14 post-estrus: PCD7 and PCD14) and the Day 7 of the first embryo transfer (1ETD7)

Upstream Regulator	Molecule Type	Predicted Activation State	PCD7		PCD14		1ETD7	
			Activation z-score	p-value of overlap	Activation z-score	p-value of overlap	Activation z-score	p-value of overlap
AGT	growth factor	Activated	3.63	4.26E-05	3.13	3.28E-04	4.31	1.55E-04
CD38	enzyme	Activated	2.77	1.31E-02	2.43	3.59E-02	2.81	1.50E-02
Cigarette smoke	chemical toxicant	Activated	2.40	8.47E-04	2.22	2.26E-02	2.77	3.05E-03
cyclic AMP	chemical - endogenous mammalian	Activated	2.37	2.46E-03	2.24	2.26E-02	2.13	3.05E-03
EGR2	transcription regulator	Activated	2.18	4.31E-03	2.77	3.31E-03	2.47	1.48E-03
F2	peptidase	Activated	3.71	1.04E-03	3.22	3.44E-03	3.28	4.44E-04
HGF	growth factor	Activated	3.73	1.82E-02	2.89	4.22E-03	3.15	1.14E-02
Hydrogen peroxide	chemical - endogenous mammalian	Activated	3.88	1.58E-02	3.79	3.21E-03	2.61	4.90E-03
IFNG	cytokine	Activated	2.92	2.15E-04	4.52	7.41E-05	3.30	1.54E-05
IL1A	cytokine	Activated	3.09	2.44E-02	2.08	3.76E-02	2.74	4.39E-05
IL1B	cytokine	Activated	2.47	1.25E-03	2.06	1.19E-03	3.03	1.17E-06
IL2	cytokine	Activated	2.75	4.31E-02	3.59	3.57E-03	3.16	2.23E-05
IL5	cytokine	Activated	3.22	2.59E-02	3.14	1.32E-02	3.38	1.33E-02
Lipopolysaccharide	chemical drug	Activated	3.72	5.08E-03	4.22	2.81E-04	6.07	8.69E-12
miR-155-5p (miRNAs w/seed UAAUGCU)	mature microRNA	Inhibited	-2.92	5.04E-03	-2.81	3.84E-03	-2.76	1.77E-02
miR-16-5p (and other miRNAs w/seed AGCAGCA)	mature microRNA	Inhibited	-2.91	2.36E-02	-2.78	1.65E-02	-3.09	1.05E-02
NFkB (complex)	complex	Activated	2.82	8.40E-03	2.98	2.13E-02	4.12	1.35E-03
PD98059	chemical - kinase inhibitor	Inhibited	-3.10	3.09E-05	-2.14	2.53E-04	-3.75	4.75E-05
TNF	chemical drug	Activated	3.77	2.91E-02	2.55	5.38E-05	4.21	2.17E-06
Vegf	enzyme	Inhibited	-2.24	1.89E-04	2.70	4.63E-07	2.94	8.52E-03
VEGFA	chemical drug	Inhibited	-3.36	6.06E-04	2.19	8.07E-04	2.48	8.07E-03

Validation of selected genes in WBCs by RT-qPCR relatively to pregnancy outcome

Twelve (12) WBCs expressed genes (*AIF1, DCK, FNDC3B, GLUL, IL15, IFI27L2, MYADM, PTGR1, SERPINE1, SOX5, TCN2* and *VASH1,*) were selected (Table 3) and their expression was quantified using quantitative real time PCR (RT-qPCR).

Table 3: Primer sequences used for real-time PCR (RT-qPCR) quantification of transcript levels of a selection of 12 differentially expressed genes (DEGs) and 3 housekeeping genes (*ACTB, GAPDH, RPL19*)

Gene Name	Unigene / GeneID	Accession	Forward (For.)and reverse(Rev.) primers sequences(5'->3)	Product length (bp)
AIF1	ID:280989	NM_173985	For:TCTGCCATTCTAAAAATGATCCTG<br>Rev:CCTCACCACAAATCAGGGCA	114
DCK	ID:530642	NM_001034573	For:GAGCCACTCCAGAGAAATGCTT<br>Rev:TTCTATGCAGGAGCCAGCTTT	123
FNDC3B	ID:615534	NM_001206211	For:GAGGAGACCCTCACTGCCTA<br>Rev:GGCCTGAATTCTGATCCGGT	198
GLUL	ID:281199	NM_001040474	For:GGTTAACAGGTGGAAGCCCA<br>Rev:GGCCTGCTTTAGTGACATGC	184
IFI27L2	ID:535465	NM_001076061	For:TCCAGTCCGTGGGGGC<br>Rev:TGGAGGAGGGGAAGGAGATG	119
IL15	ID:281248	NM_174090	For:CAGCGATGCAGTGCTTTCTC<br>Rev:TCCTCACATTCTTTGCATCCCA	157
MYADM	ID:506295	NM_001075252	For:CTGACAGGCATCAACCTGCT<br>Rev:TGAGGGACACAGACAAGGGA	228
PTGR1	ID:513177	NM_001035281	For:ATCGCTAAGCTCAAGGGCTG<br>Rev:CGGGAGCAGCTTCTTTCAGA	145
SERPINE1	ID:281375	NM_174137	For:CTCCGAGGTTTGAGACCCAC<br>Rev:ACACCTTGTCTCTCCTTGCG	157
SOX5	ID:533829	NM_001083471	For:ATAGAGAATCCAGGGGGCGT<br>Rev:CTTTCCAGCGAGATCCCAGT	159
TCN2	ID:281518	NM_174195	For:CTCCAGACACCCCGGTAATG<br>Rev:TGAGTGACCTTCTCGGGACA	203
VASH1	ID:615419	NM_001206803	For:AGACCTCTGACAGGGCTGAT<br>Rev:CACGATGTGGCGGAAGTAGT	177
**Housekeeping genes**				
ACTB	ID:280979	NM_173979.3	For:GAATCCTGCGGCATTCACGA<br>Rev:GCGCGATGATCTTGATCTTCATT	186
GAPDH	ID:281181	NM_001034034.2	For:GCCGATGCCCCCATGTTTGT<br>Rev:TCATAAGTCCCTCCACGATGC	150
RPL19	ID:510615	NM_001040516.2	For:CCCAATGAGACCAATGAAATC<br>Rev:CAGCCCATCTTTGATCAGCTT	73

These genes were selected based on their differential expression between heifers classified as non-pregnant and those classified as pregnant at each time point, with two additional heifers included in the non-pregnant group (n = 9). Of the 12 candidate genes, 7 have been validated by RT-qPCR. Thus *PTGR1* (Prostaglandin reductase 1, FC = 4.8) mRNA abundance was significantly greater in WBCs from the non-pregnant group compared to those from the pregnant group at PCD7, PCD14, and 1ETD7 (Fig 7). *GLUL* (FC = 1.34) transcript was significantly higher ($p \leq 0.05$) at PCD7 or exhibited a trend toward a higher expression (p = 0.054) in the heifers of the non-pregnant group than the heifers of the pregnant group at PCD14, (Fig 7). *AIF1* (FC = 1.99) *SERPINE1 (FC = 1.76) and MYADM (FC = 1.93)* mRNA levels were significantly higher ($p \leq 0.05$) or exhibited a trend toward a higher expression (p = 0.14; p = 0.15) in the heifers of the non-pregnant group at PCD14, respectively (Fig 7). The mRNA expression of *FNDC3B (*FC = 1.31), *IL15 (FC = 1.44)* and *SERPINE1* were significantly higher ($p \leq 0.05$) or exhibited a trend toward a higher expression (p = 0.06) in the heifers of the non-pregnant group at 1EDT7, respectively (Fig 8).

Fig 8. Gene expression in peripheral white blood cells (WBCs) of Holstein Friesian heifers during the preconception cycle and on the day of embryo transfer of the conception cycle. On Day 7 (PCD7) and on Day 14 (PCD14) of the preconception cycle as well as on Day 7 of the first conception cycle just before embryo transfer (1ETD7), expression levels of *AIF1, FNDC3B, GLUL, IL15, PTGR1* and *SERPINE1* transcripts were measured by RT-qPCR between females classified as non-pregnant (n = 9) and those classified as pregnant (n = 4). The horizontal line denotes the median value, the box encompasses the upper and lower quartiles, whiskers show the range, and the plus symbol denotes the mean. *Repeated Measures (RM) two-way ANOVA or Mixed-effects Model with Geisser-Greenhouse correction, matched values stacked into a subcolumn, Sidak's multiple comparisons test with individual variances computed for each comparison was carried out.* The results of this test are denoted in the plot with horizontal bars and asterisks (*P < 0.05; ** P< 0.01).

DISCUSSION

The establishment of a successful pregnancy following embryo transfer depends on reciprocal and synchronized interactions between the developing embryo and the maternal endometrium. The quality of an embryo, defined by its potential to develop to term, has been extensively studied using robust and relevant parameters (39). Similarly, predicting the capacity of recipient females to support a pregnancy to term, resulting in the birth of healthy offspring, has been a subject of research. In cattle, omics-based phenotyping of endometrial biopsies has identified promising biomarkers, but their routine application remains challenging (40). Consequently, identifying minimally invasive biomarkers to reliably predict pregnancy potential in recipient females without endometrial tissue sampling represents a critical challenge with significant commercial applications in reproductive management.

In this study, analyses were performed on two groups of heifers, classified as follows: (i) heifers that successfully carried a pregnancy to term after the first embryo transfer following an initial preconception cycle, including one heifer that was confirmed pregnant twice on Day 18, and classified as pregnant; and (ii) heifers that failed to establish pregnancy after two consecutive embryo transfers, classified as non-pregnant. Compared with heifers classified as pregnant, heifers classified as non-pregnant exhibited higher circulating progesterone concentrations on days 7 and 14 post-estrus during the preconception cycle. These findings have aligned with earlier studies reporting decreased conception rates in animals with elevated plasma progesterone levels (41). However, these results alone are insufficient to predict a heifer's likelihood of pregnancy or pregnancy outcomes, underscoring the need to explore additional markers. Peripheral white blood cells (WBCs) have been shown to secrete various factors in response to steroid hormones, making them a potential source of maternal biomarkers for predicting gestational success (42). A literature review suggests that evaluating peripheral lymphocyte balance, cytokine profiles, and specific interleukins could serve as predictive

biomarkers of implantation before embryo transfer in women (43). Research has also explored the identification of potential circulating biomarkers for recurrent miscarriage and/or recurrent implantation failure as novel diagnostic and predictive tools (44). However, to our knowledge, this approach has not yet been investigated in cattle in the context of embryo transfer.

In this study, our data indicate that gene expression profiles in WBCs during the estrous cycle and at the time of embryo transfer may predict implantation potential and pregnancy outcomes in Holstein Friesian heifers. Transcriptomic analyses revealed that heifers classified as non-pregnant exhibited overexpression of genes associated with immune and inflammatory responses, potentially indicating an exacerbation of the inflammatory state and activation of immune responses. In contrast, heifers classified as pregnant showed a regulated innate immune response linked to pregnancy maintenance. Increased expression of pro-inflammatory cytokine genes including *IL15,* as well as genes associated with chronic inflammation (*SERPINE1, AIF1*) and immune response (*FNDC3B*) was observed in non-pregnant females. These findings suggest that immune dysregulation during the estrous cycle may impair uterine receptivity and hinder pregnancy establishment in Holstein heifers. This aligns with studies showing that cytokine networks influence endometrial receptivity and embryo implantation (45).

A significant upregulation of Allograft Inflammatory Factor 1 (*AIF1*) transcripts was observed in WBCs from heifers classified as non-pregnant on day 14 post-estrus of the preconception cycle. AIF1, a cytokine implicated in chronic allograft rejection (46) and inflammatory processes, may reflect an inadequate maternal systemic environment for embryo implantation and pregnancy progression. Similarly, *IL15* transcript levels were significantly elevated in WBCs from heifers classified as non-pregnant at the time of embryo transfer. In humans, elevated IL-15 levels have been linked to implantation failure and recurrent spontaneous

abortions (47) and exacerbated inflammation is known to reduce conception rates in dairy cattle (5).

In the group of non-pregnant heifers, increased expression of *SERPINE1*, encoding plasminogen activator inhibitor-1 (PAI-1), further supports the hypothesis of impaired blood coagulation and fibrinolytic activity affecting pregnancy establishment. In humans, elevated PAI-1 levels are associated with recurrent pregnancy losses and other reproductive disorders (48, 49). Our findings are consistent with a previous work that identified white blood cell genes associated with the infertile phenotype in heifers, with the *SERPINE2* gene believed to play a role in heifer infertility (50).

Interestingly *PTGR1* transcripts were elevated in non-pregnant heifers throughout the preconception cycle and at the day of embryo transfer. PTGR1, a key enzyme in arachidonic acid metabolism, has been shown to play roles in inflammation and antioxidant responses (51) PTGR1 was found to be overexpressed in several cancer cell lines, including hepatocellular carcinoma, lung cancer, prostate cancer and bladder cancer, suggesting an oncogenic role for PTGR1(52). While its role in reproduction remains unexplored, its upregulation may contribute to a global profile of altered inflammation and antioxidant responses linked to pregnancy failure.

Pregnant heifers showed elevated expression of genes related to cell differentiation, lipid metabolism, skeletal mineralization, and embryonic development on Days 7 and 14 post-estrus, both during the preconception cycle and on the day of embryo transfer (Day 7 post-estrus of the conception cycle) including *CLEC1B*, *COL26A1*, *MYH14*, and *WDR31*, that were previously known as markers of uterine receptivity in cattle (25). Additionally, genes associated with the major histocompatibility complex (*BOLA-N*), calcium and potassium transport and binding (*CACNG6, KCNH2, S100G*), skeletal mineralization (*ALPL*), actin-based cell shape

and motility (*SYNPO, MYO10, MKL1*), cell cycle regulation (*PPP1R1C*), embryonic morphogenesis (*LRIG3*), and cell differentiation and organ development (*KRBA1*) showed increased expression in WBCs from pregnant heifers, supporting with the hypothesis that theses genes are involved in promoting endometrial receptivity and pregnancy success. Our results align with those of Salilew-Wondim D et al., who demonstrated that expression levels of solute carrier cluster genes, as well as genes involved in cell proliferation and the cell division cycle, were elevated in the receptive endometrium of heifers on day 7 of the estrous cycle." (53).

Additionnaly, the results of our study indicated that upstream regulators played a key role in modulating the expression of these genes. The upstream regulators miR-16-5p (and other miRNAs with the seed sequence AGCAGCA) and miR-155-5p (miRNAs with the seed sequence UAAUGCU) were predicted to be inhibited in heifers classified as non-pregnant. Previous research has reported the involvement of miRNAs from peripheral white blood cells in heifers able to conceive (54). The identification of up-stream regulators linked to inflammatory and immune responses supports that the immune system may be modulated at the peripheral level to prepare the maternal organism for pregnancy. Moreover, in our study, GO and KEGG term/functions analyses identified pathways with regulation of genes related to leukocytes transendothelial migration and inflammation response in non-pregnant animals.

Elevated mRNA expression of gene coding pro-inflammatory cytokines in WBCs from heifers classified as non-pregnant compared with those classified as pregnant may suggest that responses to molecular and cellular events creating a favorable maternal environment for new pregnancy escape the uterus and induce peripheral response. Alternatively, it could also be that the local changes reflect systemic events. These findings underscore the role of the systemic immune environment in facilitating the endometrial receptivity necessary for pregnancy. Our

results indicated that transcript levels of WBCs genes which differed at PCD7 and 1ETD7 between heifers classified as non-pregnant and pregnant, were distinct. This suggests that the gene expression profile in WBCs fluctuates across cycles, and therefore, gene expression from one cycle may not reliably predict the pregnancy outcome of the subsequent cycle upon embryo transfer.

Regulatory mechanisms must take place to protect the conceptus from rejection by the maternal immune system (55). Transcriptomic analyses performed on endometrial samples collected during the estrous cycle in heifers have shown that genes associated with inflammation, growth factors, and immune responses are indicative of receptive and non-receptive endometrium and are crucial for the establishment of pregnancy (53).

Overall, our data underline that gene expression profiles in WBCs throughout the estrous cycle could be reflective of inflammatory, immune and physiology states that may be detrimental for embryo implantation and development in heifers. The complex relationship between inflammation and pregnancy success has been well documented in humans. While regulated inflammation is crucial for embryo implantation, an exacerbated inflammatory response in immune cells may compromise the successful establishment of pregnancy (56).

This study underlined the increased expression of genes involved in potassium / calcium signaling, metabolic pathway, tissue remodeling, and positive regulation of angiogenesis in WBCs from animals of the pregnant group as well as in the endometrium supports the link between the systemic environment and the capacity of the endometrium to support pregnancy (57). This work is in keeping with a published study conducted on Angus-Simmental heifers, that were sampled at weaning and retrospectively classified as fertile or subfertile based on pregnancy diagnosis. Differentially expressed transcripts in PBMCs between the two groups

were detected, including those encoding potassium channels and genes associated with inflammation and immunity (14).

Taken together our findings support the concept of a two-way dialogue between the endometrium and peripheral white blood cells, emphasizing the importance of systemic and local immune regulation in embryo development and pregnancy establishment. Endometrial receptivity and the ability of a heifer to deliver a healthy offspring must be considered in an integrated manner. Our data offer new perspectives in the context of monitoring female fertility and prompt the need for further studies that will aim to use whole peripheral blood as a whole or specific subtypes of circulating immune cell types as sources of biomarkers to predict pregnancy issue in mammalian females.

Conclusion

Pregnancy success involves a finely coordinated series of molecular and cellular events at both local and systemic levels. Our current study demonstrates that variations in the transcriptomic abundance of WBCs genes throughout the estrous cycle could serve as a predictive tool for assessing the recipient female's capacity to support embryonic development to term after embryo transfer. Gene signatures from WBCs may offer a valuable practical approach to predict embryo transfer success in recipient mammalian females. Further large-scale studies will be necessary to validate and expand upon the results of this study.

Acknowledgments

The authors thank Dr. David G. E. Smith for his involvement in the iPUD project, Philippe Bolifraud for his technical support with sample processing, Prof. François Vialard for his helpful feedback on the manuscript, and Vincent Mauffré for valuable scientific discussions. We are also grateful to the Cellulaire Microarray Platform (IGBMC – Institute of Genetics, Molecular and Cellular Biology, Strasbourg, France; https://www.igbmc.fr/plateformes-technologiques/genomeast) for carrying out the microarray hybridizations, and to the @Bridge Genomic Analysis Platform (INRAE, Ile-de-France – Jouy-en-Josas – Antony Research Center, France) for providing access to the IPA software.

References

1. Mamo S, Mehta JP, Forde N, McGettigan P, Lonergan P. Conceptus-endometrium crosstalk during maternal recognition of pregnancy in cattle. Biol Reprod. 2012;87(1):6, 1-9.

2. Manjari P, Reddi S, Alhussien M, Mohammed S, De S, Mohanty AK, et al. Neutrophil gene dynamics and plasma cytokine levels in dairy cattle during peri-implantation period. Veterinary immunology and immunopathology. 2016;173:44-9.

3. Dickinson SE, Griffin BA, Elmore MF, Kriese-Anderson L, Elmore JB, Dyce PW, et al. Transcriptome profiles in peripheral white blood cells at the time of artificial insemination discriminate beef heifers with different fertility potential. BMC Genomics. 2018;19(1):129.

4. Fujiwara H. Do circulating blood cells contribute to maternal tissue remodeling and embryo-maternal cross-talk around the implantation period? Mol Hum Reprod. 2009;15(6):335-43.

5. Velazquez MML, Peralta MB, Angeli E, Stassi AF, Gareis NC, Durante L, et al. Immune status during postpartum, peri-implantation and early pregnancy in cattle: An updated view. Anim Reprod Sci. 2019;206:1-10.

6. Mansouri-Attia N, Oliveira LJ, Forde N, Fahey AG, Browne JA, Roche JF, et al. Pivotal role for monocytes/macrophages and dendritic cells in maternal immune response to the developing embryo in cattle. Biol Reprod. 2012;87(5):123.

7. Roberts RM, Leaman DW, Cross JC. Role of interferons in maternal recognition of pregnancy in ruminants. Proc Soc Exp Biol Med. 1992;200(1):7-18.

8. Bazer FW, Burghardt RC, Johnson GA, Spencer TE, Wu G. Interferons and progesterone for establishment and maintenance of pregnancy: interactions among novel cell signaling pathways. Reprod Biol. 2008;8(3):179-211.

9. Fujiwara H. Immune cells contribute to systemic cross-talk between the embryo and mother during early pregnancy in cooperation with the endocrine system. Reprod Med Biol. 2006;5(1):19-29.

10. Fukui A, Fujii S, Yamaguchi E, Kimura H, Sato S, Saito Y. Natural killer cell subpopulations and cytotoxicity for infertile patients undergoing in vitro fertilization. Am J Reprod Immunol. 1999;41(6):413-22.

11. Mansouri-Attia N, Sandra O, Aubert J, Degrelle S, Everts RE, Giraud-Delville C, et al. Endometrium as an early sensor of in vitro embryo manipulation technologies. Proc Natl Acad Sci U S A. 2009;106(14):5687-92.

12. Bauersachs S, Ulbrich SE, Zakhartchenko V, Minten M, Reichenbach M, Reichenbach HD, et al. The endometrium responds differently to cloned versus fertilized embryos. Proc Natl Acad Sci U S A. 2009;106(14):5681-6.

13. Shirasuna K, Matsumoto H, Kobayashi E, Nitta A, Haneda S, Matsui M, et al. Upregulation of interferon-stimulated genes and interleukin-10 in peripheral blood immune cells during early pregnancy in dairy cows. J Reprod Dev. 2012;58(1):84-90.

14. Banerjee P, Diniz WJS, Hollingsworth R, Rodning SP, Dyce PW. mRNA Signatures in Peripheral White Blood Cells Predict Reproductive Potential in Beef Heifers at Weaning. Genes (Basel). 2023;14(2).

15. Ideta A, Sakai S, Nakamura Y, Urakawa M, Hayama K, Tsuchiya K, et al. Administration of peripheral blood mononuclear cells into the uterine horn to improve pregnancy rate following bovine embryo transfer. Anim Reprod Sci. 2010;117(1-2):18-23.

16. Yu N, Yang J, Guo Y, Fang J, Yin T, Luo J, et al. Intrauterine administration of peripheral blood mononuclear cells (PBMCs) improves endometrial receptivity in mice with embryonic implantation dysfunction. Am J Reprod Immunol. 2014;71(1):24-33.

17. Okitsu O, Kiyokawa M, Oda T, Miyake K, Sato Y, Fujiwara H. Intrauterine administration of autologous peripheral blood mononuclear cells increases clinical pregnancy rates in frozen/thawed embryo transfer cycles of patients with repeated implantation failure. J Reprod Immunol. 2011;92(1-2):82-7.

18. Farifteh F, Fazeli E, Zeinab Hosseini S, Soheila Arefi S, Moini A, Taheripanah R, et al. Intrauterine administration of autologous peripheral blood mononuclear cells regulates the endometrium estrogen and progesterone receptor expression: An RCT. Int J Reprod Biomed. 2023;21(4):343-54.

19. Horka P, Jarosova R, Malickova K, Janatkova I, Mareckova H, Zima T, et al. Intracellular cytokine production in peripheral blood lymphocytes: a comparison of values in infertile and fertile women. Am J Reprod Immunol. 2011;65(5):466-9.

20. Croy BA, He H, Esadeg S, Wei Q, McCartney D, Zhang J, et al. Uterine natural killer cells: insights into their cellular and molecular biology from mouse modelling. Reproduction. 2003;126(2):149-60.

21. Pate JL, Landis Keyes P. Immune cells in the corpus luteum: friends or foes? Reproduction. 2001;122(5):665-76.

22. Paria BC, Reese J, Das SK, Dey SK. Deciphering the cross-talk of implantation: advances and challenges. Science. 2002;296(5576):2185-8.

23. Bauersachs S, Ulbrich SE, Gross K, Schmidt SE, Meyer HH, Einspanier R, et al. Gene expression profiling of bovine endometrium during the oestrous cycle: detection of molecular pathways involved in functional changes. J Mol Endocrinol. 2005;34(3):889-908.

24. Forde N, Beltman ME, Duffy GB, Duffy P, Mehta JP, O'Gaora P, et al. Changes in the endometrial transcriptome during the bovine estrous cycle: effect of low circulating progesterone and consequences for conceptus elongation. Biol Reprod. 2011;84(2):266-78.

25. Mazzoni G, Pedersen HS, Rabaglino MB, Hyttel P, Callesen H, Kadarmideen HN. Characterization of the endometrial transcriptome in early diestrus influencing pregnancy status in dairy cattle after transfer of in vitro-produced embryos. Physiol Genomics. 2020;52(7):269-79.

26. Riesewijk A, Martin J, van Os R, Horcajadas JA, Polman J, Pellicer A, et al. Gene expression profiling of human endometrial receptivity on days LH+2 versus LH+7 by microarray technology. Mol Hum Reprod. 2003;9(5):253-64.

27. Diskin MG, Waters SM, Parr MH, Kenny DA. Pregnancy losses in cattle: potential for improvement. Reprod Fertil Dev. 2016;28(1-2):83-93.

28. McMillan WH. Statistical models predicting embryo survival to term in cattle after embryo transfer. Theriogenology. 1998;50(7):1053-70.

29. Meyerholz MM, Mense K, Lietzau M, Kassens A, Linden M, Knaack H, et al. Serum IGFBP4 concentration decreased in dairy heifers towards day 18 of pregnancy. J Vet Sci. 2015;16(4):413-21.

30. Meyerholz MM, Mense K, Lietzau M, Kassens A, Linden M, Knaack H, et al. Serum IGFBP4 concentration decreased in dairy heifers towards day 18 of pregnancy. J Vet Sci. 2015;16(4):413-21.

31. Richard C, Hue I, Gelin V, Neveux A, Campion E, Degrelle SA, et al. Transcervical collection of bovine embryos up to Day 21: an 8-year overview. Theriogenology. 2015;83(7):1101-9.

32. Meyer HH, Sauerwein H, Mutayoba BM. Immunoaffinity chromatography and a biotin-streptavidin amplified enzymeimmunoassay for sensitive and specific estimation of estradiol-17 beta. J Steroid Biochem. 1990;35(2):263-9.

33. Raliou M, Dembele D, Duvel A, Bolifraud P, Aubert J, Mary-Huard T, et al. Subclinical endometritis in dairy cattle is associated with distinct mRNA expression patterns in blood and endometrium. PLoS One. 2019;14(8):e0220244.

34. Dembele D, Kastner P. Fold change rank ordering statistics: a new method for detecting differentially expressed genes. BMC Bioinformatics. 2014;15:14.

35. Smyth GK. Linear models and empirical bayes methods for assessing differential expression in microarray experiments. Stat Appl Genet Mol Biol. 2004;3:Article3.

36. Benjamini YaH, Y. Controlling The False Discovery Rate - A Practical And Powerful Approach To Multiple Testing1995 1995/11/30.

37. Lê S, Josse, J., and Husson, F. FactoMineR : An R Package for Multivariate Analysis. J Stat Softw 25. 2008;25(1).

38. Vandesompele J, De Preter K, Pattyn F, Poppe B, Van Roy N, De Paepe A, et al. Accurate normalization of real-time quantitative RT-PCR data by geometric averaging of multiple internal control genes. Genome Biol. 2002;3(7):RESEARCH0034.

39. Lonergan P. State-of-the-art embryo technologies in cattle. Soc Reprod Fertil Suppl. 2007;64:315-25.

40. Rabaglino MB, Kadarmideen HN. Machine learning approach to integrated endometrial transcriptomic datasets reveals biomarkers predicting uterine receptivity in cattle at seven days after estrous. Sci Rep. 2020;10(1):16981.

41. Nogueira MF, Melo DS, Carvalho LM, Fuck EJ, Trinca LA, Barros CM. Do high progesterone concentrations decrease pregnancy rates in embryo recipients synchronized with PGF2alpha and eCG? Theriogenology. 2004;61(7-8):1283-90.

42. Dixit VD, Parvizi N. Pregnancy stimulates secretion of adrenocorticotropin and nitric oxide from peripheral bovine lymphocytes. Biol Reprod. 2001;64(1):242-8.

43. Benkhalifa M, Joao F, Duval C, Montjean D, Bouricha M, Cabry R, et al. Endometrium Immunomodulation to Prevent Recurrent Implantation Failure in Assisted Reproductive Technology. Int J Mol Sci. 2022;23(21).

44. King K, Smith S, Chapman M, Sacks G. Detailed analysis of peripheral blood natural killer (NK) cells in women with recurrent miscarriage. Hum Reprod. 2010;25(1):52-8.

45. Orsi NM. Cytokine networks in the establishment and maintenance of pregnancy. Hum Fertil (Camb). 2008;11(4):222-30.

46. Utans U, Arceci RJ, Yamashita Y, Russell ME. Cloning and characterization of allograft inflammatory factor-1: a novel macrophage factor identified in rat cardiac allografts with chronic rejection. J Clin Invest. 1995;95(6):2954-62.

47. Chegini N, Ma C, Roberts M, Williams RS, Ripps BA. Differential expression of interleukins (IL) IL-13 and IL-15 throughout the menstrual cycle in endometrium of normal fertile women and women with recurrent spontaneous abortion. J Reprod Immunol. 2002;56(1-2):93-110.

48. Ye Y, Vattai A, Zhang X, Zhu J, Thaler CJ, Mahner S, et al. Role of Plasminogen Activator Inhibitor Type 1 in Pathologies of Female Reproductive Diseases. Int J Mol Sci. 2017;18(8).

49. Gris JC, Neveu S, Mares P, Biron C, Hedon B, Schved JF. Plasma fibrinolytic activators and their inhibitors in women suffering from early recurrent abortion of unknown etiology. J Lab Clin Med. 1993;122(5):606-15.

50. Moorey SE, Walker BN, Elmore MF, Elmore JB, Rodning SP, Biase FH. Rewiring of gene expression in circulating white blood cells is associated with pregnancy outcome in heifers (Bos taurus). Sci Rep. 2020;10(1):16786.

51. Yokomizo T, Izumi T, Takahashi T, Kasama T, Kobayashi Y, Sato F, et al. Enzymatic inactivation of leukotriene B4 by a novel enzyme found in the porcine kidney. Purification and properties of leukotriene B4 12-hydroxydehydrogenase. J Biol Chem. 1993;268(24):18128-35.

52. Wang X, Yin G, Zhang W, Song K, Zhang L, Guo Z. Prostaglandin Reductase 1 as a Potential Therapeutic Target for Cancer Therapy. Front Pharmacol. 2021;12:717730.

53. Salilew-Wondim D, Holker M, Rings F, Ghanem N, Ulas-Cinar M, Peippo J, et al. Bovine pretransfer endometrium and embryo transcriptome fingerprints as predictors of pregnancy success after embryo transfer. Physiol Genomics. 2010;42(2):201-18.
54. Banerjee P, Diniz WJS, Rodning SP, Dyce PW. miRNA expression profiles of peripheral white blood cells from beef heifers with varying reproductive potential. Front Genet. 2023;14:1174145.
55. Ott TL. Symposium review: Immunological detection of the bovine conceptus during early pregnancy. J Dairy Sci. 2019;102(4):3766-77.
56. Lin M, Xu H, Qiu J. Inflammation in recurrent miscarriage - a comprehensive perspective from uterine microenvironment and immune cell imbalance to therapeutic strategies. Ginekol Pol. 2024;95(4):266-75.
57. Moran B, Butler ST, Moore SG, MacHugh DE, Creevey CJ. Differential gene expression in the endometrium reveals cytoskeletal and immunological genes in lactating dairy cows genetically divergent for fertility traits. Reprod Fertil Dev. 2017;29(2):274-82.

SUPPORTING INFORMATION

S1 Table: Holstein Friesian Heifers involved in the experimental protocol: pregnancy phenotype and biochemical/molecular analyses.

S2 Table: DEGs in peripheral white blood cells isolated on Day 7 of preconception cycle (PCD7) from heifers classified as pregnant (n = 4) upon embryo transfer vs. non-pregnant (n = 7) at conception cycles 1 and 2.

S3 Table: DEGs in peripheral white blood cells isolated on Day 14 of preconception cycle (PCD14) from heifers classified as pregnant (n = 4) upon embryo transfer vs. non-pregnant (n = 7) at conception cycles 1 and 2.

S4 Table: DEGs in peripheral white blood cells (WBCs) isolated on Day 7 of the first conception cycle just before embryo transfer from heifers classified as pregnant (n = 4) upon embryo transfer vs. non-pregnant (n = 7) at conception cycles 1 (1ETD7).

S5 Table. Common DEGs between PCD7, PCD14 and 1ETD7 in peripheral white blood cells isolated from heifers classified as pregnant (n = 4) upon embryo transfer vs. non-pregnant (n = 7) at conception cycles 1 and 2.

S6 Table: IPA for networks associated with DEGs in peripheral white blood cells collected on PCD7 from heifers classified as pregnant (n = 4) upon embryo transfer vs. non pregnant (n = 7) at conception cycles 1 and 2.

S7 Table: IPA for networks associated with DEGs in peripheral white blood cells collected on PCD14 from heifers classified as pregnant (n = 4) upon embryo transfer vs. non-pregnant (n = 7) at conception cycles 1 and 2.

S8 Table: IPA for networks associated with DEGs in peripheral white blood cells collected on 1ETD7 from heifers classified as pregnant (n = 4) upon embryo transfer vs. non-pregnant (n = 7) at conception cycles 1 and 2.

S9 Table: Major functional categories and pathways (GO and KEEG) associated with genes significantly enriched in peripheral white blood cells of heifers classified as non-pregnant upon embryo transfer vs. non-pregnant at conception cycles 1 and 2.

S10 Table: Upstream regulators predicted to explain gene expression changes observed at PCD7 in peripheral white blood cells of heifers classified as pregnant (n = 4) upon embryo transfer vs. non-pregnant (n = 7) at conception cycles 1 and 2.

S11 Table: Upstream regulators predicted to explain gene expression changes observed at PCD14 in peripheral white blood cells of heifers classified as pregnant (n = 4) upon embryo transfer vs. non-pregnant (n = 7) at conception cycles 1 and 2.

S12 Table. Upstream regulators predicted to explain gene expression changes observed at 1ETD7 in peripheral white blood cells of heifers classified as pregnant (n = 4) upon embryo transfer vs. non-pregnant (n = 7) at conception cycles 1 and 2.

S13 Table: Common upstream regulators at PCD7, PCD14 and 1ETD7 predicted to explain gene expression changes observed in peripheral white blood cells (WBCs) of heifers classified as pregnant (n = 4) vs. non pregnant (n = 7) .

Figure 1 Click here to access/download;Figure;Fig.1.tif

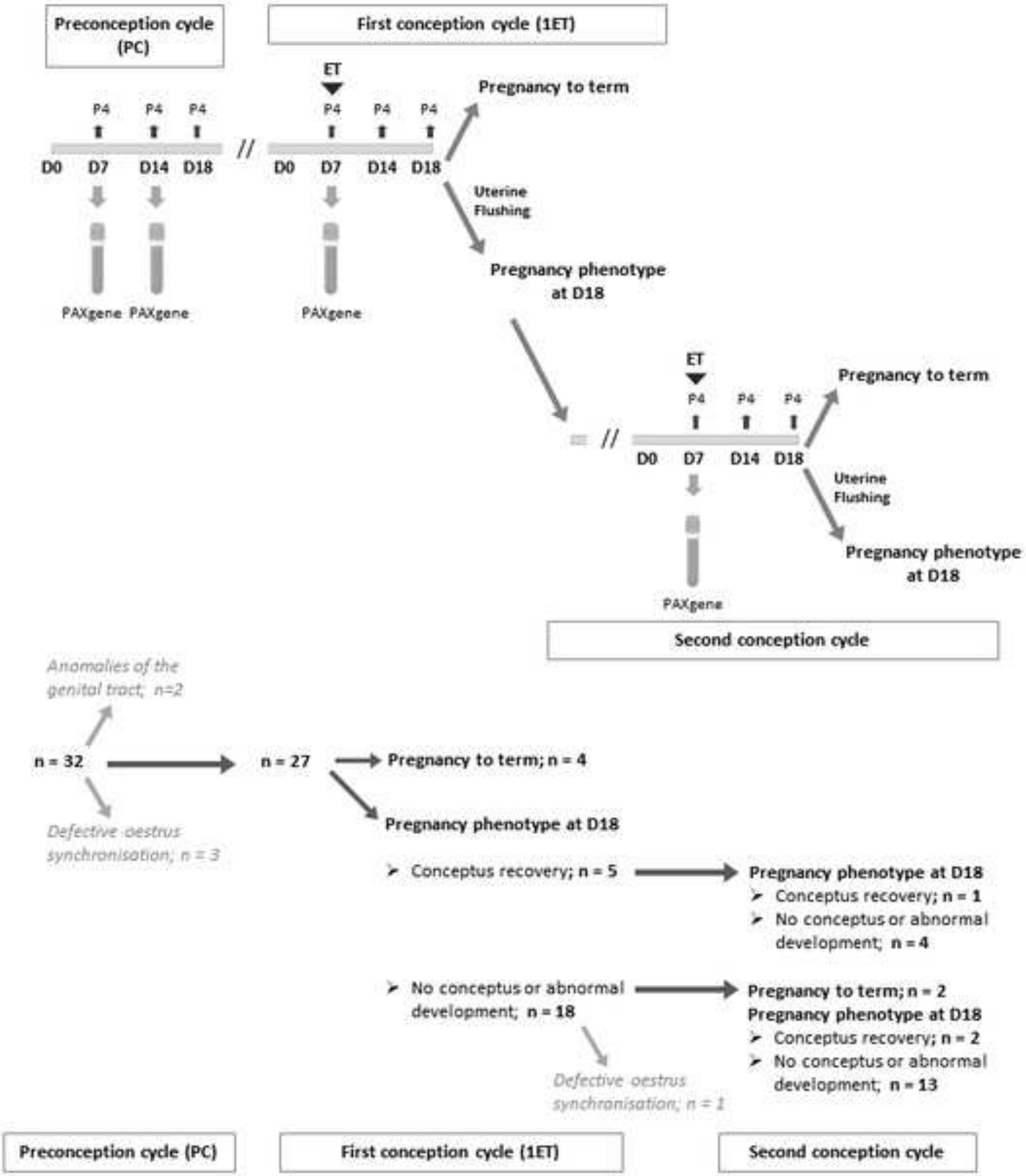

Fig 1

 Click here to access/download;Figure;Fig.2.tif

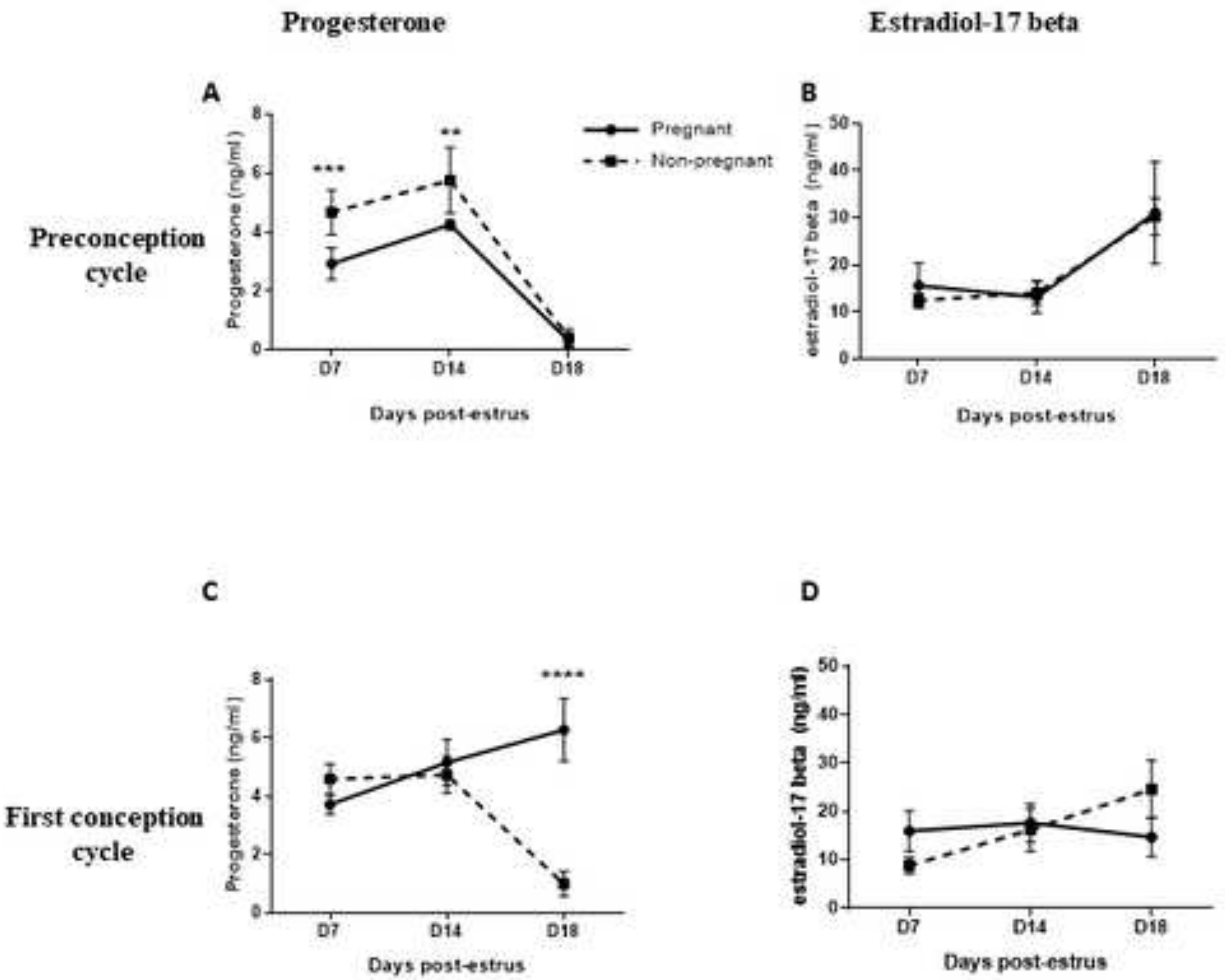

Fig 2

Figure 3

Click here to access/download;Figure;Fig.3.tif

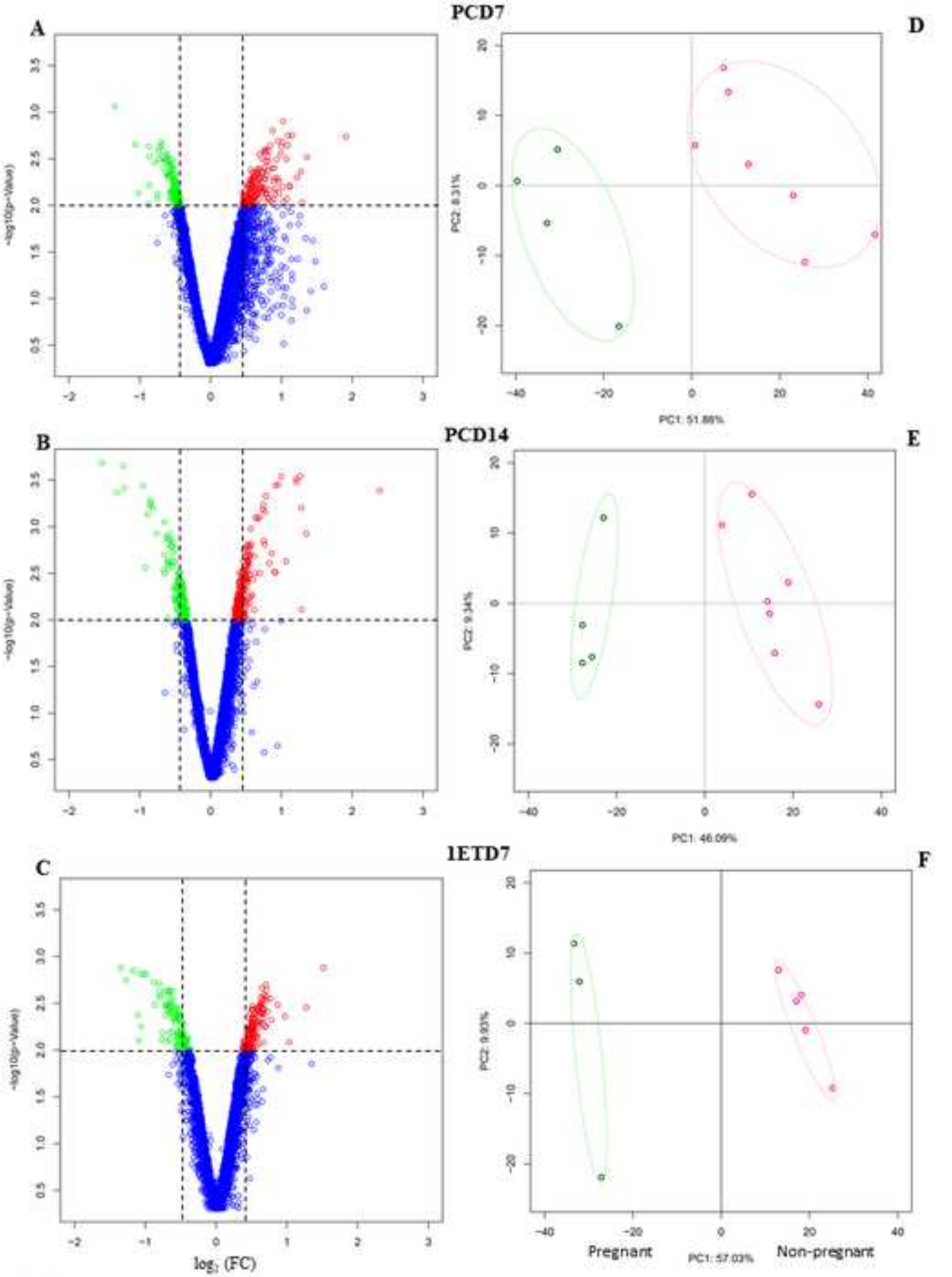

Fig 3

Figure 4 Click here to access/download;Figure;Fig.4.tif

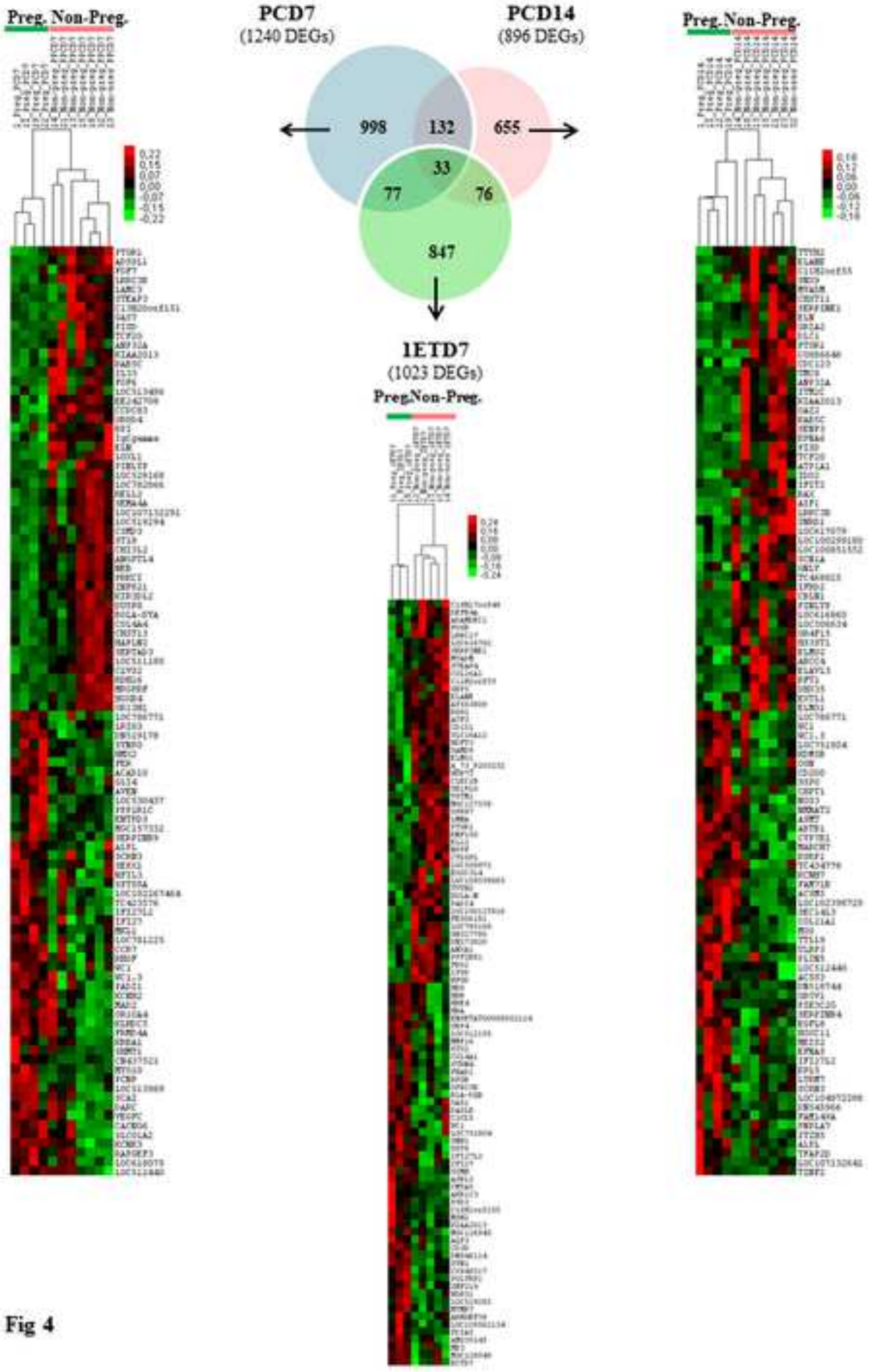

Fig 4

Click here to access/download;Figure;Fig.5.tif

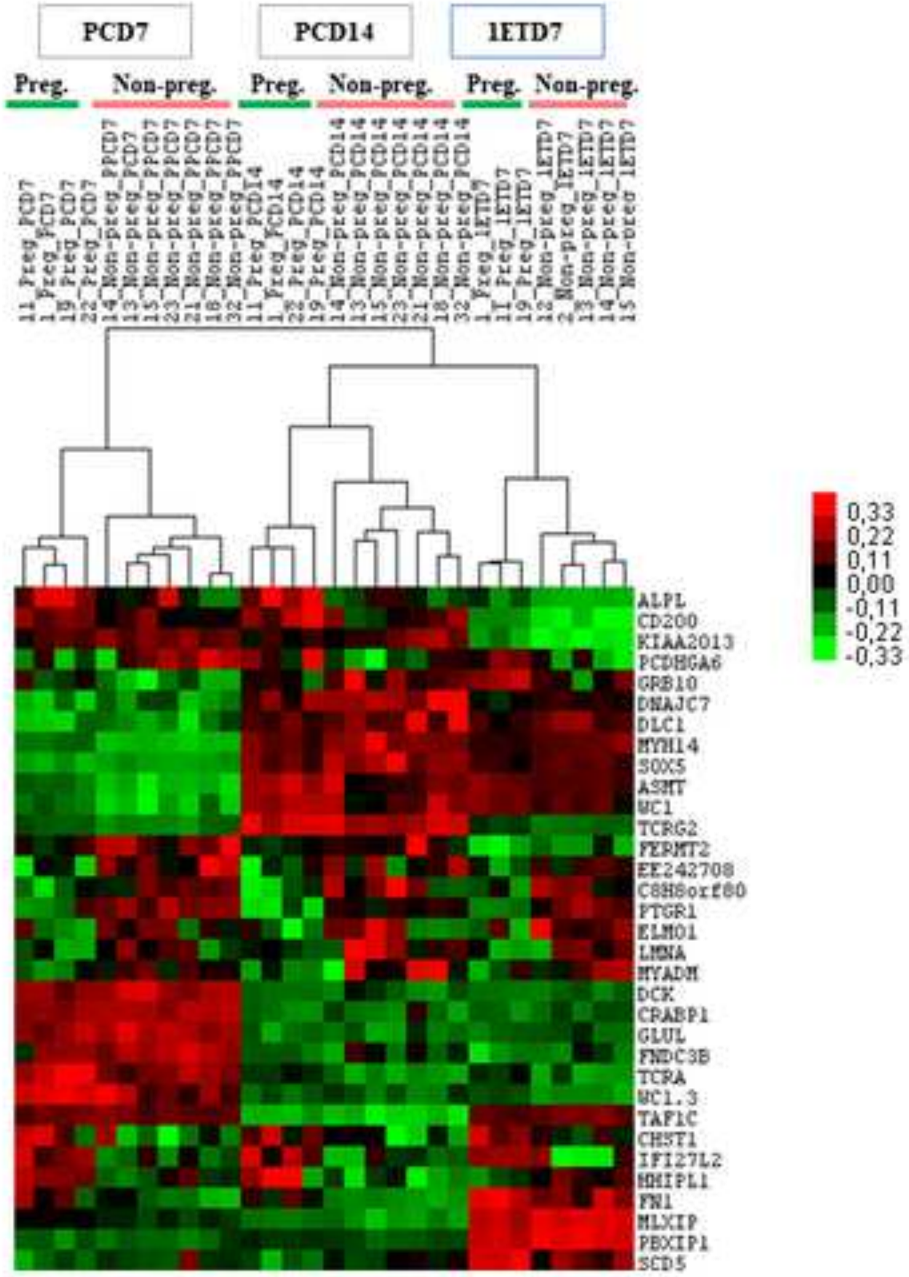

Fig 5

Figure 6

Click here to access/download;Figure;Fig.6.tif

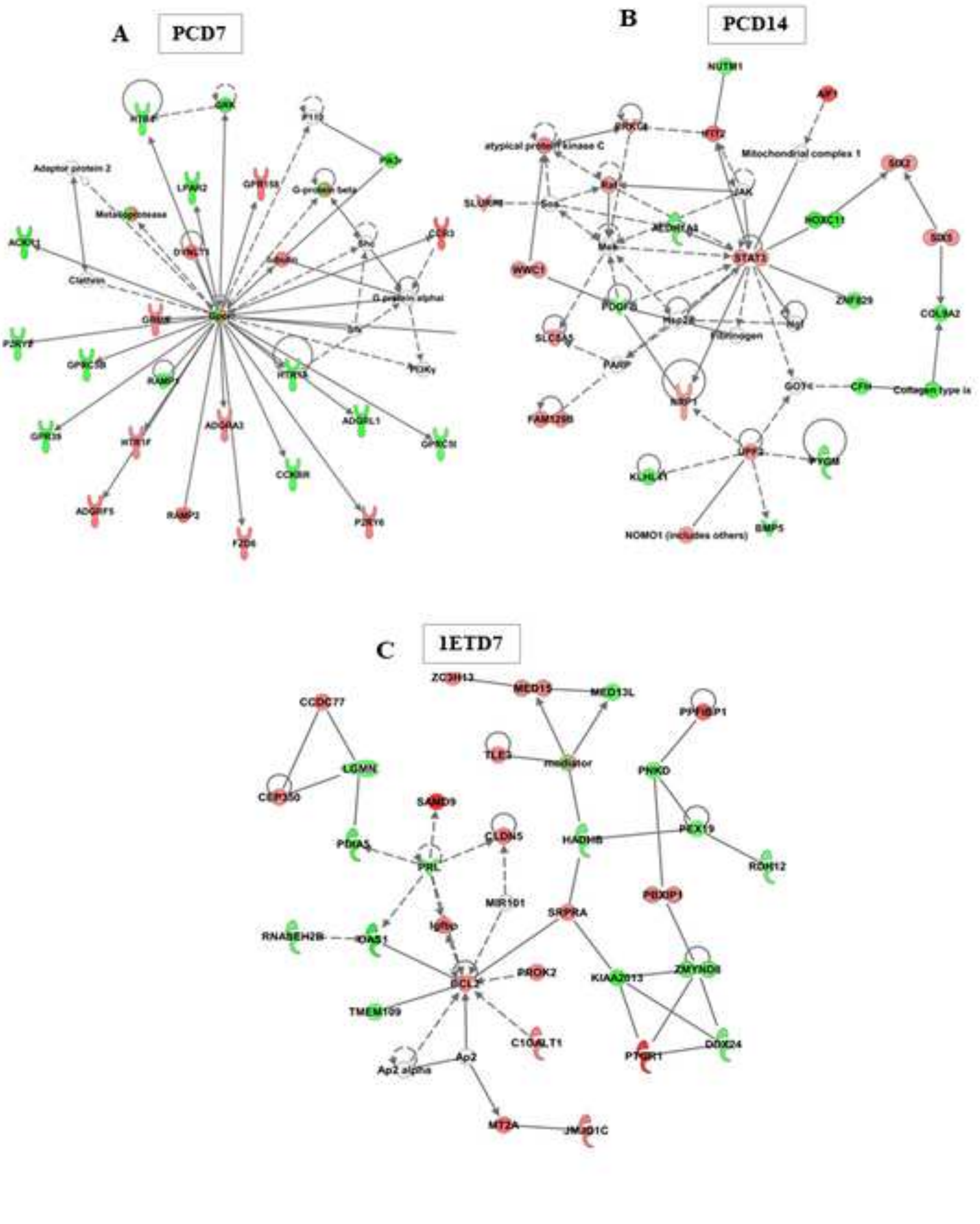

Fig 6

Figure 7 Click here to access/download;Figure;Fig.7.tif

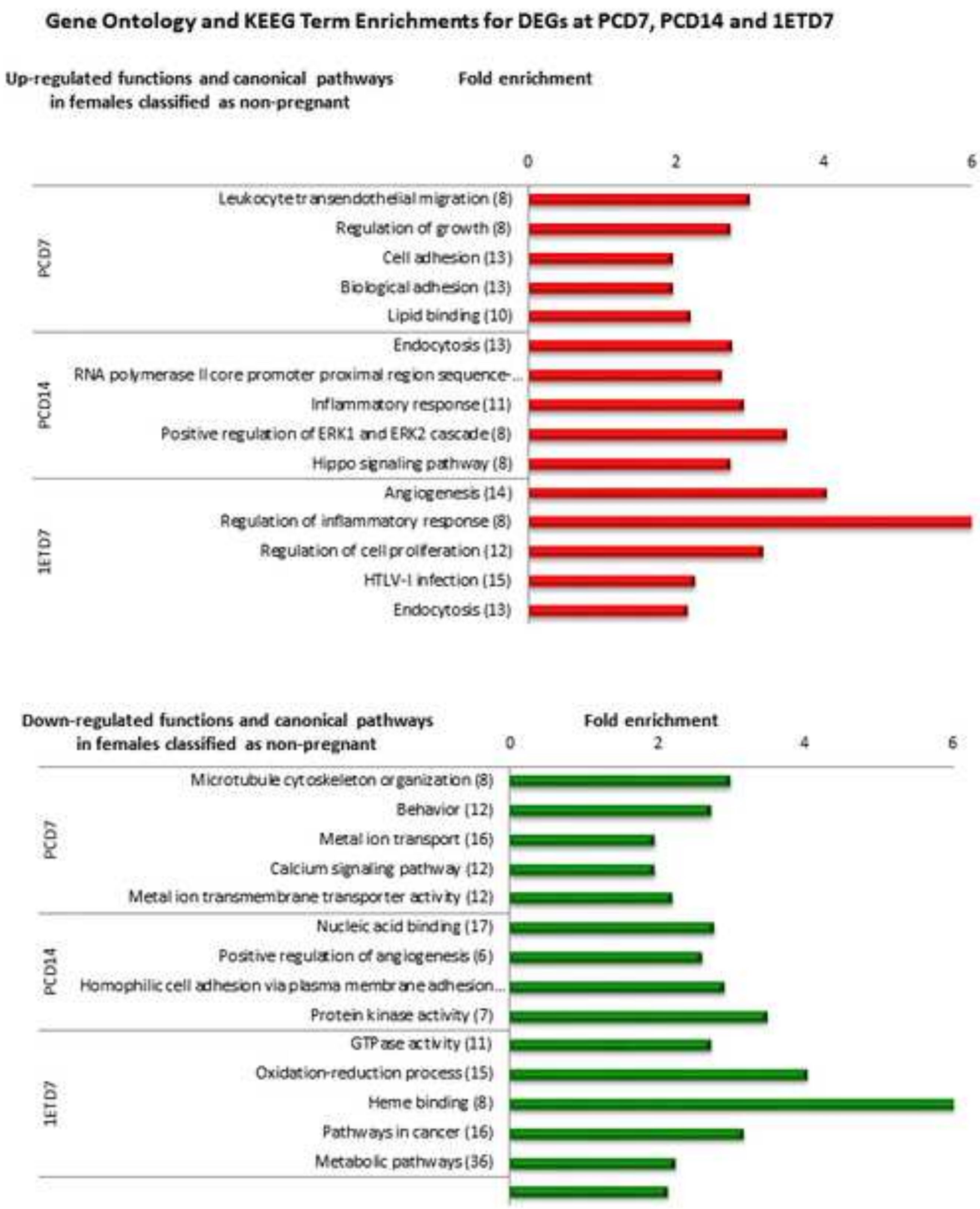

Fig 7

Figure 8

Click here to access/download;Figure;Fig.8.tif

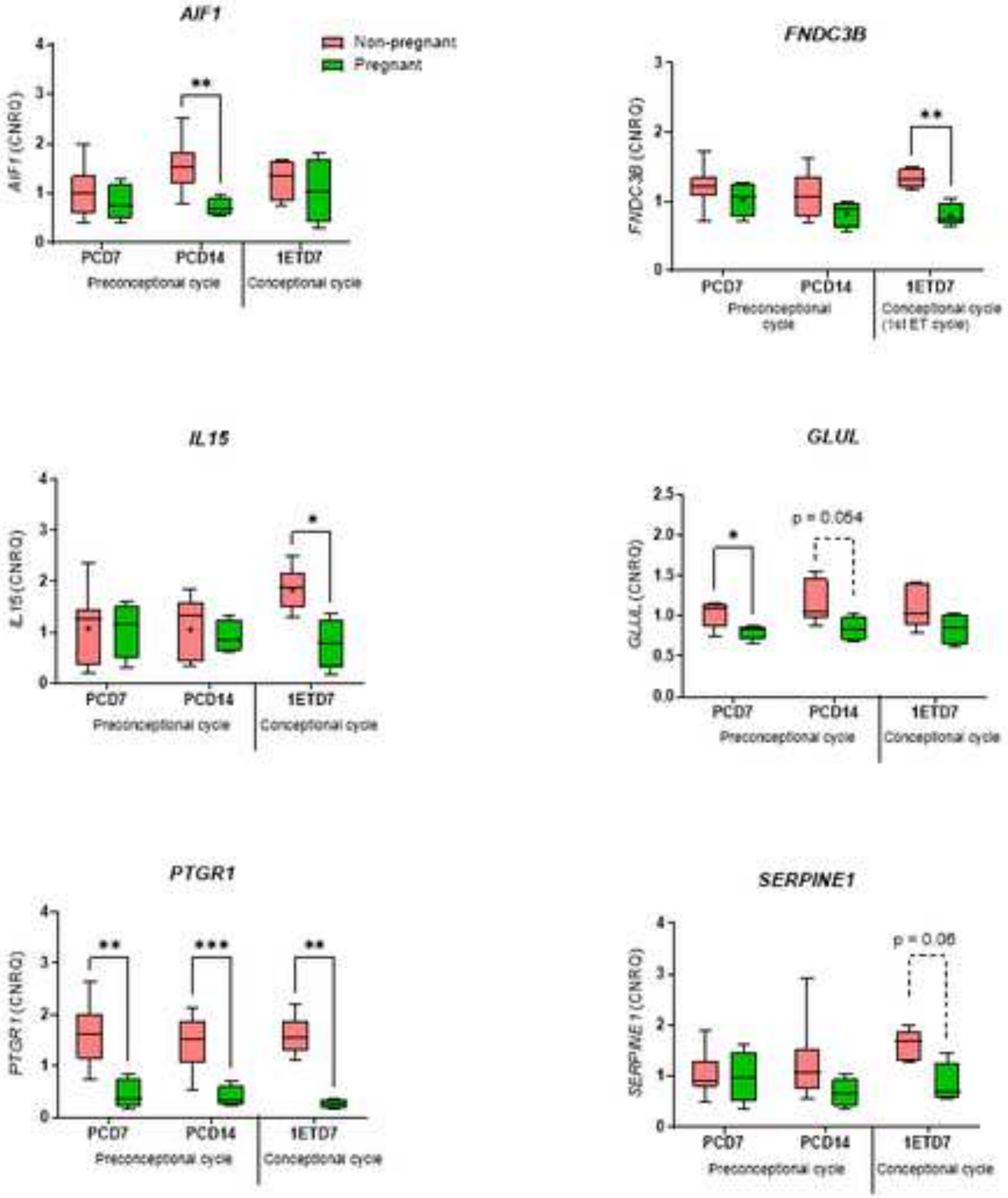

Fig 8

S1 Table

Click here to access/download
Supporting Information
S1 Table.xlsx

S2 Table

Click here to access/download
Supporting Information
S2 Table.xlsx

S3 Table

Click here to access/download
Supporting Information
S3 Table.xlsx

S4 Table

Click here to access/download
Supporting Information
S4 Table.xlsx

S5 Table

Click here to access/download
Supporting Information
S5 Table.xlsx

S6 Table

Click here to access/download
Supporting Information
S6 Table.xlsx

S7 Table

Click here to access/download
Supporting Information
S7 Table.xlsx

Click here to access/download
Supporting Information
S8 Table.xlsx

S9 Table

Click here to access/download
Supporting Information
S9 Table.xlsx

S10 Table

Click here to access/download
Supporting Information
S10 Table.xlsx

S11 Table

Click here to access/download
Supporting Information
S11 Table.xlsx

S12 Table

Click here to access/download

Supporting Information

S12 Table.xlsx

S13 Table

Click here to access/download
Supporting Information
S13 Table.xlsx
