# Supplementary material for: Gene profiles of peripheral white blood cells as potential predictors of pregnancy in embryo-recipient heifers
Source: PLoS One. 2025 Sep 15;20(9):e0330701. doi: 10.1371/journal.pone.0330701 (PMC12435678; doi:10.1371/journal.pone.0330701)
Supplement: Attachment — Submitted filename: Response to Reviewers.docx [file pone.0330701.s016.docx]

**Responding to Peer Reviewer Comments**

**Modifications and revisions of the manuscript text refer to the lines of the Revised Manuscript with Track Changes.**

**Reviewer #1**: Experiments were conducted rigorously, with appropriate controls and sample sizes.

The conclusions were appropriately presented, based on the data reported

Statistical analysis have been performed correctly and rigorously

The language in submitted articles is clear, correct, and unambiguous

The manuscript described multiple techniques and generated data that supports the conclusions.

**Authors’ Response:**

We would like to express our sincere gratitude for your thoughtful and positive feedback on our manuscript.

**Reviewer #2**: The presented investigation is highly applied in nature and is executed. The developments regarding alternative tools for diagnosing pregnancy has the potential to significantly enhance the fertility goals in the cattle industry and could pave the way as a model for analogous applications within human medicine. The experimental design, in conjunction with the statistical analyses conducted, has been well designed and carried out in a very professional manner. However, it is my considered opinion that the discussion section would greatly benefit from the inclusion of more in-depth arguments, which would effectively introduce novel ideas or suggest new directions for further exploration. In my humble opinion, this article is worthy of acceptance, albeit with the recommendation for some minor improvements to be made specifically in the discussion section to improve its overall quality. I extend my sincere gratitude to the research team for their efforts in bringing forth innovative interventions that contribute meaningfully to our understanding of this important field.

**Authors’ Response:**

We would like to thank you for your thoughtful and positive comments. Your feedback has been very useful in refining and improving the quality of our work

In response to your suggestions, we have made revisions to both the “Materials and Methods and Discussion” sections. Additionally, we made minor revisions to the manuscript to improve its clarity.

**Lines 45-53**: In the abstract, we have clarified the syntax, corrected the breed name to “Holstein-Friesian”, and replaced “oestradiol-17b with “estrogens”, as the assay kit used measures all estrogens, not just oestradiol-17b.

**Lines 78-80**: We have added a sentence to enhance the Introduction and revised the syntax to improve clarity.

**Lines 94 and 114**: We have revised the manuscript to follow the international nomenclature standards for cell populations.

**Line 108**: We have added an “s” to PBMC to indicate the plural form.

**Line 128**: We have adopted the official nomenclature of TGF-β.

**Line 138**: We have added an “s” to WBC to indicate the plural form

**Lines 199-607**: We have made minor corrections to the legends of Fig 1 (lines 199-205), Fig 2 (lines 374-375), Fig 3 (lines 414, 415 and 420), Fig 4 (lines 443-450), Fig 5 (lines 467-470), Fig 6 (lines 494-499, and 501-505), Fig 7 (lines 533-540), and Fig 8 r(lines 600-607), to improve clarity.

**Lines 262-263, 271,-272, 290, 292, 295,352, 353, 407, and 408**: Minor edits have been made to improve the sentence syntax.

**Line 248**: we have corrected the term (1EDT) to (1ETD)

**Line 297**: We have clarified the full definition of the DAVID (Database for Annotation, Visualization and Integrated Discovery) and removed the redundant instance (lines 516-517).

**Line 383**: To improve readability, we removed the term 'peripheral white blood cells' and retained only the abbreviation (WBCs), which had already been defined earlier in the manuscript.

**Lines 547, 548, 553-555, and 566-569**: We made minor revisions to clarify the syntax and the molecule types have been corrected for TNF, VEGF, and VEGFA. These corrections have also been made in Table 2, The number of upstream regulators was updated from 21 to 24 (line 548). Consequently, the upstream regulators Tetradecanoylphorbol acetate, TGFB1, and U0126 were added to Table 2.

**Lines 572** **and 573**: We have clarified the title of Table 2 by adding « days » and « conception cycle prior to ».

**Line 578**: We made minor revisions to clarify the syntax.

**Line 580**: The words 'quantitative real-time' have been removed to avoid repetition, as RT-qPCR already implies this.

**Line 582-583**: We have removed the reference genes, *ACTB, GAPDH*, and *RPL19* from the table title, as they are already specified within the table: The term “Housekeeping genes” was replaced by “reference genes” in both the title and the content of Table 3.

**Line 588**: ‘Thus” was removed to improve the clarity of the sentence.

**Line 589**: We have added (p ≤ 0.05) to specify the threshold for the p-value applied.

**Lines 590, 593** and **595**: The numbering of Figure 8 has been corrected.

**Lines 1066-1116**: Minor clarifications have been made to the supporting table legends.

To improve the clarity and ensure consistency with the internationally accepted name of the breed, we have replaced "Friesian Holstein" with "Holstein-Friesian" throughout the manuscript, including the in the legends of figures, tables, and supporting tables.

We hope this information satisfies the journal’s requirements and are happy to provide any further clarification if needed.

**Journal Requirements**

Journal Requirements:

When submitting your revision, we need you to address these additional requirements.

1. Please ensure that your manuscript meets PLOS ONE's style requirements, including those for file naming. The PLOS ONE style templates can be found at <https://journals.plos.org/plosone/s/file?id=wjVg/PLOSOne_formatting_sample_main_body.pdf> and <https://journals.plos.org/plosone/s/file?id=ba62/PLOSOne_formatting_sample_title_authors_affiliations.pdf>

**Authors’ Response:** We would like to thank you for your thorough review of our manuscript titled “**Gene profiles of peripheral white blood cells as potential predictors of pregnancy in embryo-recipient heifers**” authored by Raliou et al. We have updated our manuscript according to the instructions provided in the PLOS ONE style templates.

2. To comply with PLOS ONE submissions requirements, in your Methods section, please provide additional information regarding the experiments involving animals and ensure you have included details on methods of anesthesia and/or analgesia.

**Authors’ Response:** Thank you for your suggestion. We would like to clarify that the animals used in this study were not euthanized. Embryo transfer and uterine flushing procedures have been revised and described in greater detail in the Materials and Methods section, specifically in lines **155-177, 182-187, and 193** and in lines **209-217, and 222**.

3. We note that the grant information you provided in the ‘Funding Information’ and ‘Financial Disclosure’ sections do not match.

When you resubmit, please ensure that you provide the correct grant numbers for the awards you received for your study in the ‘Funding Information’ section.

**Authors’ Response:** Thank you for pointing out the discrepancy between the grant information in the “Funding information” and Financial Disclosure” sections. We apologize for this oversight. We included the correct number of the grant in the “Funding information” section.

4. When completing the data availability statement of the submission form, you indicated that you will make your data available on acceptance. We strongly recommend all authors decide on a data sharing plan before acceptance, as the process can be lengthy and hold up publication timelines. Please note that, though access restrictions are acceptable now, your entire data will need to be made freely accessible if your manuscript is accepted for publication. This policy applies to all data except where public deposition would breach compliance with the protocol approved by your research ethics board. If you are unable to adhere to our open data policy, please kindly revise your statement to explain your reasoning and we will seek the editor's input on an exemption. Please be assured that, once you have provided your new statement, the assessment of your exemption will not hold up the peer review process.

**Authors’ Response:** We understand the importance of clarifying our data sharing plan and bringing it into compliance with the journal's policies. We would like to inform you that our data has already been submitted to GEO and will be made freely available as soon as the article is accepted. We will update our data availability statement to reflect this and ensure compliance with the journal's open data policy.

5. We note that you have included the phrase “data not shown” in your manuscript. Unfortunately, this does not meet our data sharing requirements. PLOS does not permit references to inaccessible data. We require that authors provide all relevant data within the paper, Supporting Information files, or in an acceptable, public repository. Please add a citation to support this phrase or upload the data that corresponds with these findings to a stable repository (such as Fig share or Dryad) and provide and URLs, DOIs, or accession numbers that may be used to access these data. Or, if the data are not a core part of the research being presented in your study, we ask that you remove the phrase that refers to these data.

**Authors’ Response:** We apologize for including « data not shown » in the manuscript, as this does not meet PLOS data sharing requirements. Since this data in question is not essential to the main findings of our study, it has been removed (**lines 360 and 361**)*.*

**Line 628-849: Discussion**

We have further clarified the classification of the group of animal group based on their phenotype following pregnancy outcome. As you recommended, we have generally improved and strengthened our discussion.

**Lines 854-860**: The conclusion has been revised and clarified to enhance its clarity and overall impact.

6. Please remove all personal information, ensure that the data shared are in accordance with participant consent, and re-upload a fully anonymized data set.

Note: spreadsheet columns with personal information must be removed and not hidden as all hidden columns will appear in the published file.

Additional guidance on preparing raw data for publication can be found in our Data Policy (https://journals.plos.org/plosone/s/data-availability#loc-human-research-participant-data-and-other-sensitive-data) and in the following article: http://www.bmj.com/content/340/bmj.c181.long.

**Authors’ response**: We have reviewed the data and removed all personal or identifying information by participant consent and ethical standards. Please note that all columns containing sensitive data have been permanently deleted, not merely hidden, to ensure compliance with PLOS publication policy. We have also reviewed the shared resources to ensure they are in accordance with participant consent, and re-upload a fully anonymized data set.

We would also like to inform you that the name of co-author **Marie Margarete Meyerholz** has been updated to **Marie Margarete Meyerholz-Wohllebe** (**line 4**) and that a new co-author, **Dr. David Smith** (Independent Microbiologist, Edinburgh, UK [smitdge@googlemail.com](mailto:smitdge@googlemail.com)) has been added to the authors list (**line 6**). Dr. David Smith contributed to the conceptualization of the study and read the manuscript. The affiliation number of each co-author has been updated accordingly (**lines 6, 7, and 20-24**)*.* **Dr. David Smith’s** name has been removed from the acknowledgments (**line 863**).

7. Please review your reference list to ensure that it is complete and correct. If you have cited papers that have been retracted, please include the rationale for doing so in the manuscript text, or remove these references and replace them with relevant current references. Any changes to the reference list should be mentioned in the rebuttal letter that accompanies your revised manuscript. If you need to cite a retracted article, indicate the article’s retracted status in the References list and also include a citation and full reference for the retraction notice.

**Authors’ response**: We have checked every bibliographical reference to ensure the list was complete and correct. None of the cited articles have been retracted to date. We have updated the reference list to reflect the modifications made to the Introduction and Discussion sections (lines 874-1065).

Although the study is quite interesting, I believe it needs to be validated through a field trial.

**Authors’ response**: We do agree with your suggestion.

A field trial will be critical to validate the robustness of our findings in real-world settings. In fact, a study involving heifers from commercial farms, under typical farming condition, is currently underway in our laboratory to further validate our current data and identify additional insights.

Could you please explain the statistical analysis and clarify whether the data is normally distributed?

Additionally, could you explain clearly what a repeated measures two-way ANOVA or a mixed-effects model entails?

**Authors’ response**:

Our data do not follow a normal distribution, and with a small sample size (n ≤ 30), normality tests lack sufficient statistical power. Therefore, we chose a two-way repeated measures ANOVA (Analysis of Variance) or Mixed-Effects Models, which are more robust to moderate deviation from normality. Given the repeated measurements for the same heifer under different conditions (non-pregnant vs. pregnant phenotype across multiple sampling days: PCD7, PCD14 and 1ETD7), these methods allow for the evaluation of the main effects and interactions while accounting for intra-individual correlation.

- The Two-way Repeated Measures ANOVA assesses the effect of two factors (phenotype and sampling day) while considering the repeated nature of measurements from the same individual. This test evaluates the main effects of each factor and their interaction.
- The Mixed-Effects Model extends this by incorporating both fixed effects (phenotype and sampling day) and random effects (accounting for individual variability and other unmeasured factors). This approach is particularly suitable for our data structure, as it models the dependence of repeated measurements within individuals and captures between-individual variability.

**Line 159**, 2alpha should be underscript throughout the manuscript

**Authors’ response**

The notation 2alpha (2α) has been formatted as a subscript (**lines 166 and 221**).

**Line 159**, please write the exact dosage like mg/ml

**Authors’ response**:

We added the exact dosages of cloprostenol (**lines 167 and 222**) and buserelin (**line 169**) and completed the kit details for the assays of progesterone and estrogens, including the coefficients of variation. We have also completed the information regarding the animal feeding conditions. These details were incorporated into **lines 155-171**, as well as into **lines 182-187**, and **line 211**.

We also detailed the information regarding the embryo transfer protocol as described in **lines 184-187, and 193** and for uterine flushing in **lines 209-217**.

Please write the complete kit details for hormones with a coefficient of variation.

**Authors’ response**: Thank you for raising this point.

The kit details as well as the intra-assay and inter-assay coefficients of variation have been added to **lines 236-242.**

Furthermore, the assay kit used quantifies all estrogens including estradiol 17-beta. As result estradiol 17-beta was replaced with "estrogens" in **lines 51, 204, 229, 230, 234, 244, 371** and **374** as well as in **Table 1** and **Fig 2**.

Please provide complete details of the real-time quantitative PCR (qPCR) analysis.

**Authors’ response**: We have added details of the real-time quantitative PCR (qPCR) analysis (**lines 302-338**)

**Line 328**, f-value is not the standard terminology, pls write it scientifically.

**Authors’ Response:** The microarray data were analyzed using the Fold Change Rank Ordering Statistics (FCROS) as described by (34) and t-test with the TREAT option of the LIMMA methods (7). FCROS does not rely on the concept of False Discovery Rate (FDR). Instead, FCROS is based on ranking fold change values across all pairwise comparisons between condition and control samples. For each gene, a mean rank score (f-value) is computed. Since FCROS is a non-parametric method that does not assume any underlying theoretical distribution, it does not require multiple testing correction such as FDR.

We have previously used the FCROS statistical method to analyze transcriptomic data from DNA microarray in our paper by Raliou M et al. published in 2019 in PLOS ONE (doi: 10.1371/journal.pone.0220244. eCollection 2019).
